# DON-Apt19S bioactive scaffold transplantation promotes *in situ* spinal cord repair in rats with transected spinal cord injury by effectively recruiting endogenous neural stem cells and mesenchymal stem cells

**DOI:** 10.1016/j.mtbio.2025.101753

**Published:** 2025-04-10

**Authors:** Bi-Qin Lai, Rong-Jie Wu, Chuang-Ran Wu, Hai-Yang Yu, Jing Xu, Shang-Bin Yang, Zheng-Hong Chen, Xing Li, Yi-Nan Guo, Yue Yang, Ming-Tian Che, Ting-Ting Wu, Guang-Tao Fu, Yu-Hui Yang, Zhen Chen, Nan Hua, Rui Liu, Qiu-Jian Zheng, Yuan-Feng Chen

**Affiliations:** a Department of Orthopedics, Guangdong Provincial People's Hospital (Guangdong Academy of Medical Sciences), Southern Medical University, Guangzhou, China; b Key Laboratory for Stem Cells and Tissue Engineering, Sun Yat-sen University, Ministry of Education, Guangzhou, China; c Guangdong Provincial Key Laboratory of Brain Function and Disease, Zhongshan School of Medicine, Sun Yat-sen University, Guangzhou, China; d Shantou University Medical College, Shantou, China; e Co-innovation Center of Neuroregeneration, Nantong University, Nantong, China; f Department of Histology and Embryology, Zhongshan School of Medicine, Sun Yat-sen University, Guangzhou, China; g Institute of Spinal Cord Injury, Sun Yat-sen University, Guangzhou, China; h Rehabilitation Medicine Department, The First Affiliated Hospital of Sun Yat-sen University, Guangzhou, Guangdong, China; i Medical Research Institute, Guangdong Provincial People's Hospital (Guangdong Academy of Medical Sciences), Southern Medical University, Guangzhou, China; j State Key Laboratory of Dampness Syndrome of Chinese Medicine, Department of Orthopedic Surgery, The Second Affiliated Hospital of Guangzhou University of Chinese Medicine, Guangzhou, China; k Cedars Sinai Biobank & Research Pathology Resource, Cedars-Sinai, Los Angeles, CA, 90048, USA; l National Engineering Research Center for Healthcare Devices, Guangdong Key Lab of Medical Electronic Instruments and Polymer Material Products, Institute of Biological and Medical Engineering, Guangdong Academy of Sciences, Guangzhou, China

**Keywords:** Spinal cord injury, *In situ* repair, Endogenous stem cells, Neurogenesis, Revascularization

## Abstract

The spinal cord's limited regeneration is attributed to the scarcity of endogenous stem cells and a poor post-injury microenvironment in adult mammals. To overcome these challenges, we transplanted a DNA aptamer 19S (Apt19S) sustained-release decellularized optic nerve (DON) scaffold (DON-A) into completely transected spinal cord injury (SCI) site in rats and investigated its effect on endogenous stem cell recruitment and differentiation, which subsequently contributed to *in situ* SCI repair. It has been demonstrated that Apt19S specifically binds to the membrane receptor alkaline phosphatase highly expressed on neural stem cells (NSCs) and mesenchymal stem cells (MSCs), and our study further proved that Apt19S can simultaneously recruit endogenous NSCs and MSCs to the lesion of SCI. In our study, the DON-A promoted stem cell proliferation in the early stage of the injury, followed by the rapid neurogenesis through NSCs and revascularization via MSCs. Synaptic connections between corticospinal tracts and calcitonin gene-related peptide positive nerve fibers with newborn neurons confirmed the formation of endogenous neuronal relays at the injury site, which improved the rats' motor and sensory functions. This study offers a new strategy for recruiting both NSCs and MSCs to synergistically overcome low spinal cord self-repair ability, holding a high potential for clinical translation.

## Highlights

• DON-A recruited endogenous NSCs and MSCs efficiently by specifically binding to ALPL via sustained Apt19S release.
• DON-A created CNS regenerative microenvironment for neurogenesis and revascularization in the spinal cord.
• DON-A reconstructed both the ascending and descending neural pathways in injured spinal cord.
• DON-A transplantation improved the rats' motor and sensory functions.

## Graphical abstract

In this study, we developed and transplanted a bioactive DON-A scaffold for *in situ* spinal cord repair. DON-A ensured sustained Apt19S release and can selectively recruit endogenous NSCs and MSCs into the CNS regenerative microenvironment created by DON-A, where they rapidly initiated tissue regeneration and repair, causing neural pathways reconstruction and revascularization in the spinal cord.

**Figure:**
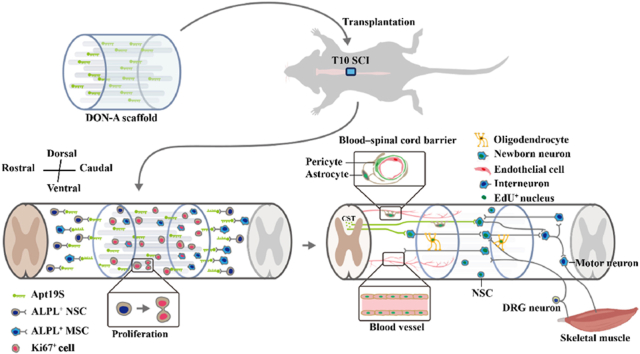

## 1 Introduction

From traditional perspective, severe spinal cord injury (SCI) lack self-repair capacity because of hypoxic ischemia and an inflammatory microenvironment, which are not conducive to regeneration [[1], [2], [3]], as well as the absence of endogenous stem cells, which results in the inability to replenish lost cells [4,5]. Recent studies have revealed that the inflammatory cytokines produced during the acute phase of SCI have positive and negative effects [2,6,7]. For instance, although inflammatory cytokines can cause necrosis and nerve cell apoptosis, they also activate endogenous stem cells that are beneficial for tissue repair [1,2,5]. The activation and committed differentiation of endogenous stem cells have the potential to overcome stem cell transplantation challenges, such as immune rejection, poor long-term survival, and it therefore holds great potential for clinical translation [2,5,8].

Recent studies indicate that the first two weeks after SCI are critical for the activation and proliferation of endogenous neural stem cells (NSCs) [2,5,9]. However, the NSCs that are activated by inflammatory cytokines often undergo apoptosis or differentiate into astrocytes, which aggravates scarring in the inflammatory microenvironment [7,10]. Li et al. transplanted stromal cell-derived factor 1 (SDF-1) sustained-release collagen scaffolds into the lesions in rats with SCI, which successfully recruited endogenous NSCs [3]. Furthermore, studies have shown that biomaterials with neurotrophic factors or mesenchymal stem cells (MSCs) exosomes enhance the survival of endogenous NSCs and promote their differentiation into neurons or oligodendrocytes for SCI repair [[11], [12], [13]]. Thus, strategies that recruit endogenous NSCs while preventing their apoptosis and promoting committed differentiation are critical for SCI repair [1,4,14]. Although peripheral blood MSCs can be used as a source of endogenous MSC to participate in SCI repair [15]. However, no studies have reported drugs or bioactive materials can be used for the simultaneous recruitment of both endogenous NSCs and peripheral blood-derived MSCs for SCI repair.

The DNA aptamer 19S (Apt19S), discovered in recent years, is a small molecule nucleic acid drug that can specifically bind to the alkaline phosphatase (ALPL) receptor on the membrane of stem cells [16,17]. Apt19S is reported to specifically recruit stem cells with high ALPL expression, including embryonic and pluripotent stem cells [17,18]. In recent years, Apt19S has been found to specifically recruit MSCs and NSCs, making it an outstanding candidate for therapy in cartilage and neurodegenerative disease [16,19,20]. However, the effect of Apt19S on stem cell recruitment in the CNS injury is still unknown. In this study, we loaded Apt19S into a carboxyl-rich decellularized optic nerve (DON) scaffold by exploiting the ability of amino-modified Apt19S (Apt19S-NH2) to specifically bind to protein carboxyl groups, and developed a bioactive DON-A scaffold capable of persistently releasing Apt19S with the degradation of DON. Our previous studies have shown that DON contains uniformly distributed longitudinal channels to guide nerve fiber regeneration and small pores on the channel wall for cellular and neurite communication. Importantly, proteomic analysis suggests that the extracellular matrix of DON is similar to embryonic optic nerve [21,22], which can provide physical support and suitable developmental microenvironment for endogenous stem cells recruited by Apt19S to achieve *in situ* SCI repair. Therefore, this study investigated the mechanisms underlying the recruitment and “incubation” of endogenous NSCs and MSCs via bioactive DON-A, which achieves *in situ* nerve regeneration and revascularization following SCI. Given that there are already clinical application of porcine-derived decellularized scaffolds, the transplantation of DON-A scaffolds hold great promise to provide a new strategy for the treatment of clinical SCI by recruiting endogenous stem cells to achieve *in situ* SCI repair.

## 2 Materials and methods

### 2.1 Apt19S preparation

Apt19S was synthesized by Shanghai Sangon Biotech Cooperation (China) and purified using high-performance liquid chromatography (Agilent, Japan) on a C-18 column. The aptamer sequences are as follows:

1) Apt19S: 5′-AGGTCAGATGAGGAGGGGGACTTAGGACTGGGTTTATGACCTATGCGTG-3’.
2) Amino-modified Apt19S (Apt19S-NH2):5′-NH2-(A)9-AGGTCAGATGAGGAGGGGGACTTAGGACTGGGTTTATGACCTATGCGTG-3’.
3) Amino-modified Fluorescein Isothiocyanate-Apt19S (FITC-Apt19S-NH2):5′-NH2-(A)9-AGGTCAGATGAGGAGGGGGACTTAGGACTGGGTTTATGACCTATGCGTG-FITC-3’.

### 2.2 Preparation and characterization of DON and DON-A scaffold

The preparation of DON was described in our previous study [21,22]. Briefly, fresh optic nerves were extracted from approximately two-year-old pigs, supplied by Northwest Agriculture and Forestry University, China. The nerves were then placed in centrifuge tubes and rinsed overnight with double-distilled water containing 1 % penicillin-streptomycin (Gibco, Gaithersburg, MD, USA), gentamicin (100 μg/mL), and amphotericin (2.5 μg/mL, Gibco) at 4 °C. The nerves were decellularized by incubating them with 3 % Triton X-100 (Amresco, Solon, OH, USA), 4 % sodium deoxycholate (Sigma–Aldrich, St. Louis, MO, USA), DNase (50 U/mL, Sigma–Aldrich), and RNase (10 μg/mL, Sigma–Aldrich) on a shaker (Allsheng OS-100, Hangzhou, China). Next, the DONs were submerged in sterile double-distilled water in 1.5 mL centrifuge tubes and freeze-dried for 24 h. The lyophilized DON was then stored at −30 °C until use. Scanning electron microscopy (SEM) was used to evaluate the transverse and longitudinal structures of the DON scaffolds.

The Apt19S-NH2 or amino-modified FITC-labeled Apt19S (FITC-Apt19S-NH2) were combined with the DON scaffold as previously described [23]. Briefly, a 200-μm thick DON slice (3 mm wide and 4 mm long) or a DON scaffold (3 mm in diameter and 2 mm in height) was incubated in 10 mL of morpholinoethanesulfonic acid (MES, 0.1 M, pH = 6) for 30 min, at room temperature. To activate the carboxyl groups on the surface of the DON, 80 mg of 1-ethyl-3-3-[dimethylaminopropyl] carbodiimide and hydrochloride (EDC, J&K) and 60 mg of N-hydroxysuccinimide (NHS, J&K) were added into the MES solution and incubated for 20 min. The activation buffer was then removed and the activated DON was washed three times with sterile D-hanks. Next, one DON scaffold or five DON slices were incubated in 1 mL of D-hanks containing 2 nmol of Apt19S-NH2 or FITC-Apt19S-NH2 at room temperature, for 12 h, on a reciprocating oscillator. The DON-A scaffold was then washed thrice with sterile D-hanks.

To confirm successful Apt19S loading, the DON slices loaded with FITC-Apt19S or with FITC-Apt19S-NH2 were examined under a fluorescent microscope (DM6B, Leica Microsystems, Inc., Wetzlar, Germany) at an excitation wavelength of 488 nm.

### 2.3 Isolation and culture of NSCs and MSCs

NSCs were isolated from Sprague-Dawley (SD) rats, as described previously [21]. Briefly, postnatal day one rats were overly euthanized with anesthesia. The hippocampus was then separated from the brain and cultured in Dulbecco's Modified Eagle Medium (DMEM)/F12 (1:1) containing 1× B27 (Life Technologies, USA) and 20 ng/mL of basic fibroblast growth factor (bFGF, Life Technologies, USA). After 5–7 days of culture, the cells grew into neurospheres in suspension Nestin immunoreactivity was confirmed in all the neurospheres.

To isolate MSCs, postnatal day seven SD rats were overly euthanized with anesthesia, followed by tibia and femur dissection. The tibia and femur were then flushed with DMEM (supplemented with 10 % fetal bovine serum [FBS]) to collect the bone marrow. The obtained bone marrow cells were cultured in the same media at 37 °C, 5 % CO_2_ with media changed after 48 h and every three days thereafter. Passage 3–5 cells were used in subsequent experiments. The phenotype of the MSCs was confirmed via CD73 and CD90 immunoreactivity.

### 2.4 The specific binding of Apt19S to ALPL on NSCs and MSCs

First, we evaluated the expression of potential binding sites (ALPL) for Apt19S. Briefly, 1 × 10^4^ NSCs or MSCs were seeded onto glass slides (*n* = 3) and cultured in DMEM/F12 medium containing 1 % FBS or DMEM containing 10 % FBS, respectively. After 24 h, the cells were fixed with 4 % paraformaldehyde for 20 min and then subjected to immunofluorescence analysis for the expression of Nestin/ALPL on NSCs and CD73/CD90/ALPL on MSCs, respectively).

To determine the capacity of Apt19S to bind to NSCs or MSCs, 1 × 10^4^ cells for each type were cultured on glass slides (*n* = 3) for 12 h and then thoroughly washed three times with sterile D-Hanks solution. They were then incubated with 300 μL of culture medium containing FITC-Apt19S (100 nM) for 8 h. They were then fixed, followed by immunofluorescence staining for Nestin and Hoechst 33342 (Hoe). Finally, the cells were examined under a confocal microscope (Dragonfly, Oxford Instruments, UK). Macrophages (RAW 264.7) were evaluated for ALPL expression and were also incubated with 100 nM of FITC-Apt19S as a control.

### 2.5 *In vitro* analysis of Apt19S release

The DON scaffolds loaded with FITC-Apt19S-NH2 or FITC-Apt19S (for control) were incubated at 37 °C for 30 days. 1 mL D-hanks was removed and replaced with an equal volume of D-hanks at each predetermined time point (1, 2, 3, 5, 10, 15, 20, 25, 30 days). FITC was used as a model guest molecule to monitor the release kinetics of Apt19S. The collected D-hanks was then transferred into 96-well culture plates and the level of FITC release was quantified via absorbance reading at 525 nm on a multimode plate reader (Victor Nivo 5s, PerkinElmer, USA).

### 2.6 *In vitro* analysis of NSC and MSC recruitment by Apt19S

An attachment test was used to evaluate the recruitment of NSCs and MSCs by Apt19S. For NSCs, neurospheres derived from green fluorescent protein (GFP) transgenic SD rats were digested with 0.125 % pepsin for 10 min to obtain a single-cell suspension. For MSCs, GFP positive (GFP^+^) cells were digested with 0.25 % pepsin for 3 min to obtain a single-cell suspension. Next, 5 × 10^4^ GFP ^+^ NSCs in 1 mL of DMEM/F12 or 5 × 10^4^ GFP ^+^ MSCs in 1 mL of DMEM containing 10 % FBS were seeded in 24-well plates (*n* = 4) and DON-A or DON slices were placed into each well and incubated for 12 h. To confirm that Apt19S recruited the NSCs/MSCs via ALPL receptor on cell membrane, an anti-ALPL antibody (Abmart, China) was added into the media (at a 1:100 dilution) in the 24-well plate containing the DON-A scaffold to perform an antibody competition assay. The scaffolds were then fixed with 4 % paraformaldehyde for 20 min and cell attachment was examined under a confocal microscope (Dragonfly, Oxford Instruments, UK).

The capacity of DON-A scaffolds on NSCs and MSCs migration was assessed by a Transwell system (Costar, 24-well plate, 8 μm, *n* = 4). Briefly, the neurospheres were digested with 0.125 % pepsin for 10 min to get the single-cell suspension. For MSCs, cells were digested with 0.25 % pepsin for 3 min to obtain a single-cell suspension. The membrane of the lower chamber of the Transwell system was incubated with laminin for 30min before cell seeding to improve cell attachment considering the suspension growth of NSCs. For NSCs, 2 × 10^5^ cells in 200 μL of DMEM/F12 medium were seeded into the upper chambers of the Transwell system. 600 μL of DMEM/F12 medium supplemented with 1 % fetal bovine serum (FBS, Gibco) was added to the lower chamber; for MSCs, 1 × 10^5^ cells in 200 μL of DMEM medium were seeded into the upper chambers of the Transwell system. 600 μL of DMEM medium supplemented with 10 % fetal bovine serum (FBS, Gibco) was added to the lower chamber, either without any biomaterial (Blank group), or with DON or DON-A or DON-A + anti-ALPL. After incubating for 16h, the chambers were washed with PBS for 3 times and fixed with 4 % paraformaldehyde for 20 min. After washing for another 3 times, the filters were stained with crystal violet (Beyotime Biotechnology, China) for 10 min. Three random 100x bright field images were captured for each chamber using microscopy (DM6B, Leica).

### 2.7 Assessment of cell viability on scaffolds

The cell viability of NSCs on scaffolds was quantified using propidium iodide/Hoechst33342 (PI/Hoe) staining. Firstly, 5 × 10^4^ NSCs were seeded on the 24-well plates (Blank group), DON slices and DON-A slices (*n* = 4) in 24-well plates and cultured with DMEM/F12 containing 1 %FBS for 7 days. Then the scaffolds were incubated with PI dye solution (500 nM) for 10 min at 5 % CO_2_ and 37 °C, followed by being washed with D-hanks for 3 times and then fixed with 4 % paraformaldehyde. These scaffolds were then stained with Hoe dye solution (1 μg/mL) for 10 min. Hoe^+^ cells indicate total number of cells, while PI^+^ cells indicate the number of dead cells. Images were captured from three randomly chosen fields and the percentage of live cells was calculated as follows: (number of Hoe^+^ cells-number of PI^+^ cells)/number of Hoe^+^ cells × 100 %.

A Cell Counting Kit-8 (CCK-8, DOJINDO, Japan) was also performed for the scaffolds (*n* = 4) in these 3 groups above after a culture of 7 days. Briefly, the scaffolds were transferred to a 96-well plate and 100 uL medium containing 10 % CCK-8 reagent was added, followed by incubation at 5 % CO_2_ and 37 °C for 2h. The solution from each scaffold was transferred to a new 96-well plate and was assessed using an absorbance reader (Sunrise, Tecan, Männedorf, Switzerland) at an optical density of 450 nm.

### 2.8 NSCs and MSCs differentiation *in vitro*

The neuronal, glial, and oligodendrocytic differentiation of NSCs on scaffolds was assessed using immunofluorescence staining for microtubule-associated protein 2 (Map2), glial fibrillary acidic protein (GFAP), and oligodendrocyte transcription factor 2 (Olig2). First, NSCs were seeded on the DON and DON-A longitudinal slices (5 × 10^4^*, n* = 4) in 24-well plates and cultured in DMEM/F12 containing 1 % FBS for 10 days. Next, the scaffolds were fixed with 4 % paraformaldehyde, followed by immunofluorescence staining to detect Map2 positive (Map2^+^), GFAP positive (GFAP^+^), and Olig2 positive (Olig2^+^) cells.

The potential for vascular formation by MSCs on the scaffolds was assessed using immunofluorescence staining for von Willebrand factor (VWF) and CD31. First, 5 × 10^4^ MSCs were seeded onto the DON and DON-A longitudinal slices (*n* = 4) in 24-well plates and cultured in DMEM containing 10 % FBS, 25 ng/mL vascular endothelial growth factor, and 5 ng/mL bFGF for 14 days.

### 2.9 Surgery and scaffold transplantation

Female adult SD rats (weight: 220–250 g) were supplied by the Experimental Animal Center of Sun Yat-sen University and randomly divided into three groups (*n* = 24 in each group): (1) the SCI group (no scaffold implantation at the injury), (2) the DON group (DON scaffolds implanted at the injury), and (3) the DON-A group (DON-A scaffolds implanted at the injury). All animal experiment protocols were approved by the Animal Care and Use Committee of Sun Yat-sen University and they adhered to the National Institutes of Health guidelines for the Care and Use of Laboratory Animals. Rats were anesthetized using 1 % pentobarbital sodium (25–35 mg/kg, China Pharmaceutical Shanghai Chemical Reagent Company). Subsequently, a laminectomy was performed at the T9 vertebral level, and the dura mater was vertically incised using microdissection scissors, as detailed in our previous study [24]. After achieving hemostasis, a 2-mm segment of the spinal cord was completely excised at the T10 level. DON or DON-A scaffolds (3 mm in diameter and 2 mm in height, matching the dimensions of the removed spinal cord segment) were then implanted to bridge the resultant gap. Soft tissues were subsequently sutured layer by layer with 4-0 surgical sutures. All surgical procedures were conducted in sterile conditions in an area designated for animal surgery. All rats received extensive post-surgery care, including intramuscular injection of penicillin (50,000 U/kg/d), and a painkiller 0.05 mg/kg of buprenorphine (Tianjin Pharmaceutical Research Institute Pharmaceutical Co., Ltd., Tianjin, China) for seven days, and manual emiction twice daily before their automatic micturition function was reestablished.

### 2.10 5-Ethynyl-2′-deoxyuridine (EdU) tracing of endogenous proliferative cells

EdU (Beyotime Biotechnology) was intraperitoneally administered to the rats at 50 mg/kg of body weight every 24 h from the third to the fourteenth day after the operation. EdU, which labels the proliferative cells, allowed the observation of endogenous NSC and MSC differentiation. Immunofluorescence staining for EdU was done using the BeyoClick™ EdU-488 and BeyoClick™ EdU-555 assays (Beyotime Biotechnology, China) according to the manufacturer's instructions.

### 2.11 *In vivo* analysis of NSC recruitment via Apt19S

Two weeks after SCI, immunofluorescent staining for Nestin positive (Nestin^+^) cells in the longitudinal sections of the spinal cord was used to assess NSC recruitment to the injury, as well as to determine the expression of ALPL and proliferation marker Ki-67 by the recruited Nestin^+^ cells. Details on perfusion and immunofluorescence staining are described below.

### 2.12 mRNA sequencing two weeks after SCI

Two weeks after SCI, rats from the DON and DON-A groups (three per group) were selected for spinal cord mRNA sequencing. RNA extraction was carried out and RNA integrity was confirmed using agarose gel electrophoresis, which revealed 28S:18S ratios of ≥1.5. RNA purity assessment on a NanoDrop spectrophotometer revealed OD 260/280 ratios ranging from 1.8 to 2.2. RNA quantification on a Qubit fluorometer revealed concentrations of ≥500 ng/μL. RNA-Seq was carried out by Genergy Bio-Technology Co., Ltd. (Shanghai, China).

For data analysis, raw data were first converted into sequenced reads using CASAVA (Consensus Assessment of Sequence And Variation) base calling. StringTie was used to analyze the original sequence counts of known genes. The expression levels of these genes were then determined using fragments per kilobase of transcript per million fragments mapped. The DESeq2 (Differential Expression analysis for Sequencing data, version 2‌) algorithm was used to identify differentially expressed genes in the DON vs DON-A groups, with the threshold set at |log2 (fold change [FC])| ≥ 1 and *P* ≤ 0.05.

### 2.13 Electrophysiological detection of cortical motor evoked potentials (CMEPs)

Eight weeks post-SCI, CMEPs were assessed in five rats from each group using a transcranial magnetic stimulation (TMS) instrument (Model YRD CCY1, Wuhan Yiruide Medical Equipment New Technology Co., Ltd.) [25]. For the procedure, the rats were anesthetized and then positioned on a wooden board for stability. The electrophysiological setup involved placing the recording electrode in the anterior tibialis muscle of the rat's paralyzed hind limb. Concurrently, the reference electrode was placed adjacent to the calcaneal process, while the ground electrode was inserted into the dorsal skin for effective signal grounding. The instrument stimulation output was set to 100 % intensity. The TMS coil was aligned at the center of the rat's skull to ensure precise stimulation. CMEPs were recorded once the rat had regained consciousness. For each rat, 10 separate CMEP records were acquired and expressed as average amplitude.

### 2.14 Assessment of locomotor function recovery

After surgery, the functional recovery of the rats’ hindlimbs was evaluated weekly using the open-field locomotor test. Additionally, in the eighth week after surgery, the horizontal-grid locomotor function test, which used a mirror to reflect hindlimb movement, and the inclined-grid climbing test, were conducted. The Basso, Beattie, and Bresnahan (BBB) scoring system was used to quantitatively assess voluntary movements and the ability to support body weight [26]. Two blinded independent investigators scored the BBB performance.

### 2.15 Biotinylated dextran amine (BDA) anterograde tracing

To trace corticospinal tracts (CST) regeneration in the rats, 5 μL of 10 % BDA (Invitrogen D1956; Thermo Fisher Scientific, Waltham, MA, US) was injected into ten sites in the motor cortices of each hemisphere under a Leica MZ6 dissecting stereomicroscope (Leica Microsystems, Inc., Wetzlar, Germany) [25]. At each injection site, 0.25 μL of BDA was administered at a depth of 2 mm. An additional 0.25 μL was injected 1 mm below the brain surface. The animals were sacrificed two weeks after the BDA injection for CST tracing.

### 2.16 Perfusion and immunofluorescence staining

The rats were deeply anesthetized and then intracardially perfused with physiological saline containing 0.002 % NaNO2 and 0.002 % heparin, followed by 4 % paraformaldehyde. The spinal cord was then dissected and dehydrated in 30 % sucrose for a minimum of two weeks before being cryosectioned. For immunofluorescence staining, the frozen sections and scaffolds were rinsed thrice with PBS and then incubated with 10 % goat serum for 30 min at 37 °C. They were then incubated with primary antibodies diluted in PBS-0.3 % Triton X100 at 4 °C, overnight. They were then washed thrice with PBS, incubated with secondary antibodies for 2 h at 37 °C, and then counterstained with Hoe for 10 min. The antibodies used in this study are detailed in Table S1.

### 2.17 Morphological quantification

To quantify NSCs and MSCs in the *in vitro* attachment assay, we randomly selected three areas from each scaffold (*n* = 5) and counted the number of GFP^+^/Hoe ^+^ cells. To quantify *in vitro* NSC differentiation, four areas were randomly selected from each scaffold (*n* = 4) and the neuronal, oligodendrocyitc and glial differentiation was determined by the proportion of Map2^+^, Olig2^+^ and GFAP^+^ cells to Hoe ^+^ cells. To quantify *in vitro* MSC vascular differentiation, three areas were randomly selected from each scaffold (*n* = 5), and vascular differentiation was determined by the proportion of CD31 positive (CD31^+^) and VWF positive (VWF^+^) cells to Hoe^+^ cells.

For *in vivo* quantification of betaIII-Tubulin (Tuj) positive(Tuj^+^), GFAP^+^, and VWF^+^ cells, we selected one area within 2 mm rostral/caudal to the injury/graft site or in the injury/graft sites of four horizontal sections from each rat (*n* = 5). Tuj^+^, GFAP^+^, and VWF^+^ areas were converted to an area of interest (AOI) and the pixel area of each AOI was calculated by Image J. In each image, the stained area was determined as the proportion of stained pixels out of the total number of pixels, excluding cavity areas. For *in vivo* quantification of EdU positive (EdU^+^), Nestin^+^, CD68 positive (CD68^+^), Tuj^+^, Olig2^+^, GFAP^+^, and VWF^+^ cells at the injury/graft site, four horizontal sections from each rat (*n* = 5) were selected for counting the cells that were positive for the corresponding markers. The percentages of Nestin^+^/EdU^+^, CD68^+^/EdU^+^, Tuj^+^/EdU^+^, Olig2^+^/EdU^+^, GFAP^+^/EdU^+^, and VWF^+^/EdU^+^ cells were determined based on the number of the respective double-immunopositive cells out of the total number of EdU^+^ cells.

### 2.18 Statistical analyses

All statistical analyses were performed on R studio version 4.0.4 (R Foundation for Statistical Computing) and GraphPad Prism 9.2 (GraphPad Software, San Diego, US). Data were presented as means ± standard deviation. Shapiro-Wilk normality testing was used to evaluate data distribution. Brown-Forsythe test was used to test the homogeneity of variance. Student's t-tests or Mann–Whitney's U tests were applied for comparisons between two groups according to the distribution. Differences between three sets of data were compared using the one-way analysis of variance (equal variance assumed) or the Welch one-way analysis of variance (equal variance not assumed). A least significant difference (LSD)-t (equal variance assumed) or Dunnett's T3 (equal variance not assumed) test was used for multiple comparisons between groups. For data with nonnormal distribution, nonparametric Kruskal–Wallis test was used for multiple group comparisons with Dunn's post hoc testing. *P* < 0.05 indicates statistically significant differences.

## 3 Results

### 3.1 Characterization of the DON-A scaffold

**Fig. 1:**
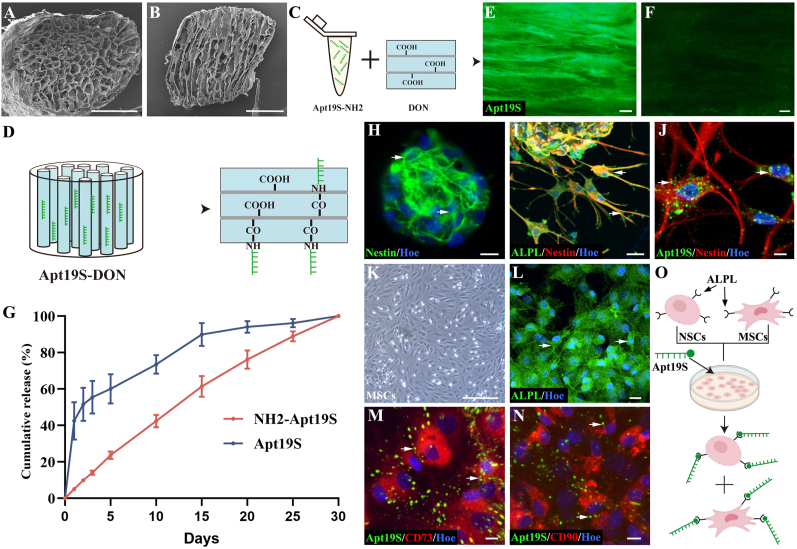
**The characteristic of DON scaffold and the specific binding of Apt19S to NSCs and MSCs *via* ALPL *in vitro*.** (A, B) Scanning electron microscopy (SEM) showing transverse (A) and longitudinal (B) structures of a DON scaffold**.** (C) Schematic diagram showing loading DON scaffold with Apt19S. (D) Schematic diagram showing Apt19S binding to DON scaffold through forming the amide bond. (E, F) Immunofluorescence image showing DON scaffold with FITC-Apt19S (E) or without any modification (F). (G) The release profile of NH2-modified Apt19S from a DON scaffold. (H) Nestin^+^ cells in a neurosphere (arrows). (I) The expression of ALPL on Nestin^+^ NSCs (arrows). (J) Immunofluorescence image showing FITC-Apt19S binds to Nestin^+^ NSCs (arrows). (K) Bright field image showing the morphology of MSCs. (L) The expression of ALPL on MSCs (arrows). (M) Immunofluorescence image showing FITC-Apt19S binds to CD73^+^ MSCs (arrows). (N) Immunofluorescence image showing FITC-Apt19S binds to CD90^+^ MSCs (arrows). (O) Schematic diagram showing Apt19S specifically binds to NSCs and MSCs via ALPL on cell membrane. Scale bars = 1 mm (A, B); 100 μm (E, F); 30 μm (I); 500 μm (K); 20 μm (L) and 10 μm (H, J, M and N).

DON was processed from fresh porcine optic nerves. Scanning electron microscopy revealed that the DON scaffold possessed uniformly distributed straight channels with uniform pore sizes, which is conducive to cell migration and neurite branch outgrowth (Fig. 1A and B). When the DON scaffold was incubated with FITC-Apt19S-NH2, the amino group in Apt19S-NH2 underwent an amidation reaction with the carboxyl group on DON, resulting in a firm bond on the scaffold surface (Fig. 1C). The longitudinal section of the DON scaffold loaded with FITC-Apt19S (DON-A) emitted green fluorescence under a fluorescence microscope (Fig. 1D and E), unlike regular DON scaffolds, which did not exhibit green fluorescence (Fig. 1F). Furthermore, the DON scaffold loaded with Apt19S (DON-A, Fig. 1G) consistently and steadily released Apt19S into the solution throughout the 30-day observation, whereas FITC-Apt19S without NH2 groups released in a burst pattern, indicating no sustained release (Fig. 1G).

### 3.2 DON-A recruits MSCs and NSCs and supports their differentiation within the microenvironment created via DON *in vitro*

To confirm the specific recruitment of NSCs and MSCs by Apt19S, we isolated NSCs from neonatal rats and cultured Nestin^+^ neurospheres (Fig. 1H). Our analysis revealed that the Nestin^+^ cytoskeleton was enveloped by ALPL^+^ cell membranes (Fig. 1I). Moreover, incubating Nestin^+^ cells with FITC-Apt19S revealed significant binding between Nestin^+^ NSCs and FITC-Apt19S on the cell surface (Fig. 1J). Analysis of cultured bone marrow MSCs, which expressed high levels of ALPL, CD73, and CD90 on the cell membrane, revealed that they could specifically bind to FITC-Apt19S (Fig. 1K-N). In order to rule out the cellular uptake of FITC-Apt19S, we co-incubated macrophages with FITC-Apt19S. The results indicated that macrophages were not labeled by FITC-Apt19S during co-incubation (Fig. S1), thus proving that FITC-Apt19S could not be uptaken even by macrophages. These results indicated the potential of Apt19S for recruiting NSCs and MSCs simultaneously via ALPL (Fig. 1O). Investigation of macrophages and vascular smooth muscle cells showed that they expressed very low ALPL levels (Fig. S2).

**Fig. 2:**
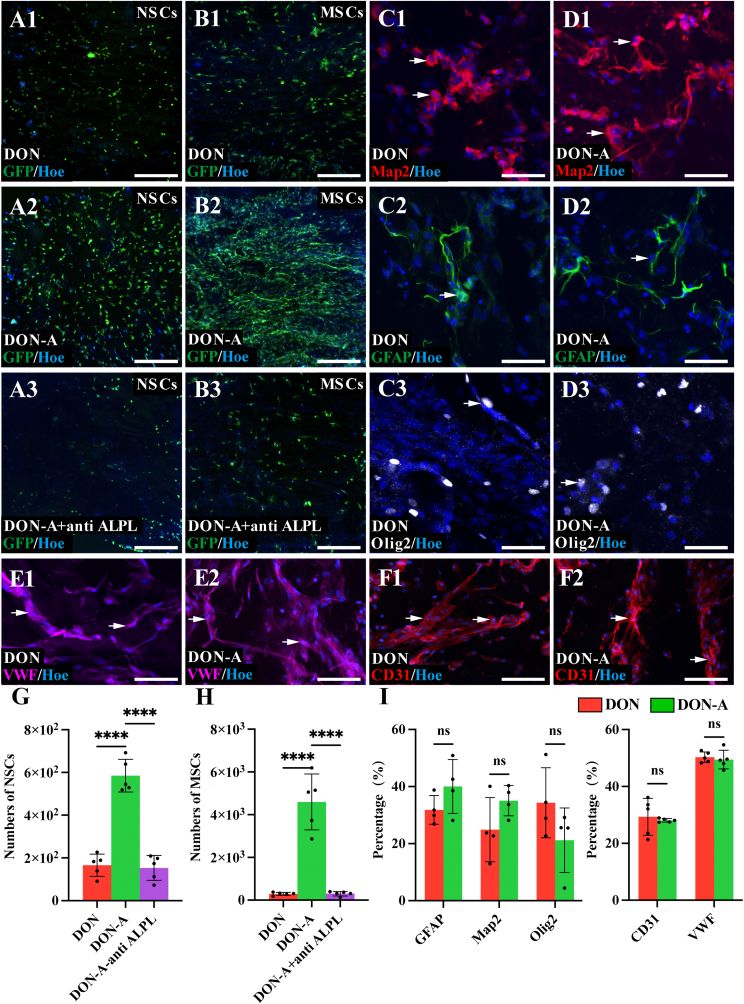
**The recruitment and the differentiation of NSCs and MSCs on DON-A scaffold.** (A1-A3) The attachment of suspended GFP^+^ NSCs onto DON scaffold (A1), DON-A scaffold (A2) and DON-A scaffold + anti-ALPL antibody (A3). (B1-B3) The attachment of suspended GFP^+^ MSCs onto DON scaffold (B1), DON-A scaffold (B2) and DON-A scaffold + anti-ALPL antibody (B3). (C1-C3) The seeded NSCs differentiating into Map2^+^ neurons (C1, arrows), Olig2^+^ oligodendrocytes (C2, arrow) and GFAP^+^ astrocytes (C3, arrow) on DON scaffolds. (D1-D3) The seeded NSCs differentiating into Map2^+^ neurons (D1, arrows), Olig2^+^ oligodendrocytes (D2, arrow) and GFAP^+^ astrocytes (D3, arrow) on DON-A scaffolds. (E1-E2) The seeded MSCs differentiating into VWF^+^ vascular endothelial cells on DON (E1, arrows) and DON-A (E2, arrows) scaffolds. (F1-F2) The seeded MSCs differentiating into CD31^+^ vascular endothelial cells on DON (F1, arrows) and DON-A (F2, arrows) scaffolds. (G, H) Bar chart showing the number of NSCs and MSCs attaching to scaffold in the three groups (*n* = 5, one-way ANOVA with LSD-t post-hoc test, ∗∗∗*P <* 0.001). (I) Bar chart showing the percentage of Map2^+^, Olig2^+^ and GFAP^+^ in all the seeded NSCs on DON and DON-A scaffolds, and the percentage of CD31^+^ and VWF^+^ in all the seeded MSCs on DON and DON-A scaffolds (*n* = 5, one-way ANOVA with LSD-t post-hoc test, ns indicates no significant difference). Scale bars = 200 μm (A1-A3, B1-B3); 20 μm (C1-C3, D1-D3, E1 -F2).

To assess the chemotactic effect of Apt19S on NSCs and MSCs, DON-A slices were cultured in 24-well plates together with NSCs or MSCs. This analysis found that the NSCs or MSCs suspended in the culture medium were more likely to adhere to DON-A scaffolds, which attracted more than threefold more cells than the blank DON scaffolds (Fig. 2A1, 2A2, 2B1 and 2B2). Moreover, adding the ALPL-blocking antibody into the cell culture medium significantly reduced the number of cells that adhered to the DON-A scaffolds, to levels similar to those observed on the blank DON scaffolds (Fig. 2A3, 2B3). To further investigate whether Apt19S promotes stem cell migration, we conducted additional Transwell test, which showed that the DON-A scaffold increased the number of NSCs and MSCs crossing the chamber, while blocking ALPL led to a decrease in the number of NSCs and MSCs crossing the chamber (Fig. S3). The *in vitro* live/dead and CCK-8 assays showed that compared to the DON group, the DON-A scaffold does not affect the survival and cell viability of NSCs (Fig. S4). Analysis of the differentiation capacity of NSCs cultured on DON-A and DON scaffolds indicated that they differentiated into Map2^+^ neurons, Olig2^+^ oligodendrocytes, and GFAP^+^ astrocytes and that their differentiation rates did not differ significantly between the two groups (Fig. 2C1-2D3). Furthermore, our findings indicate that the vascular differentiation potential of the MSCs did not differ significantly between the scaffolds, as revealed by similar rates of differentiation into VWF^+^ and CD31^+^ vascular endothelial cells (Fig. 2E1-2F2). These results confirmed that ALPL^+^ NSCs and MSCs were markedly recruited to the DON-A scaffold through the specific binding of ALPL to Apt19S (Fig. 2G and H), and indicate that Apt19S did not affect the survival, the cell viability and the normal differentiation of NSCs and MSCs (Fig. 2I).

### 3.3 DON-A specifically recruits NSCs and promotes NSC differentiation into neurons *in vivo*

**Fig. 3:**
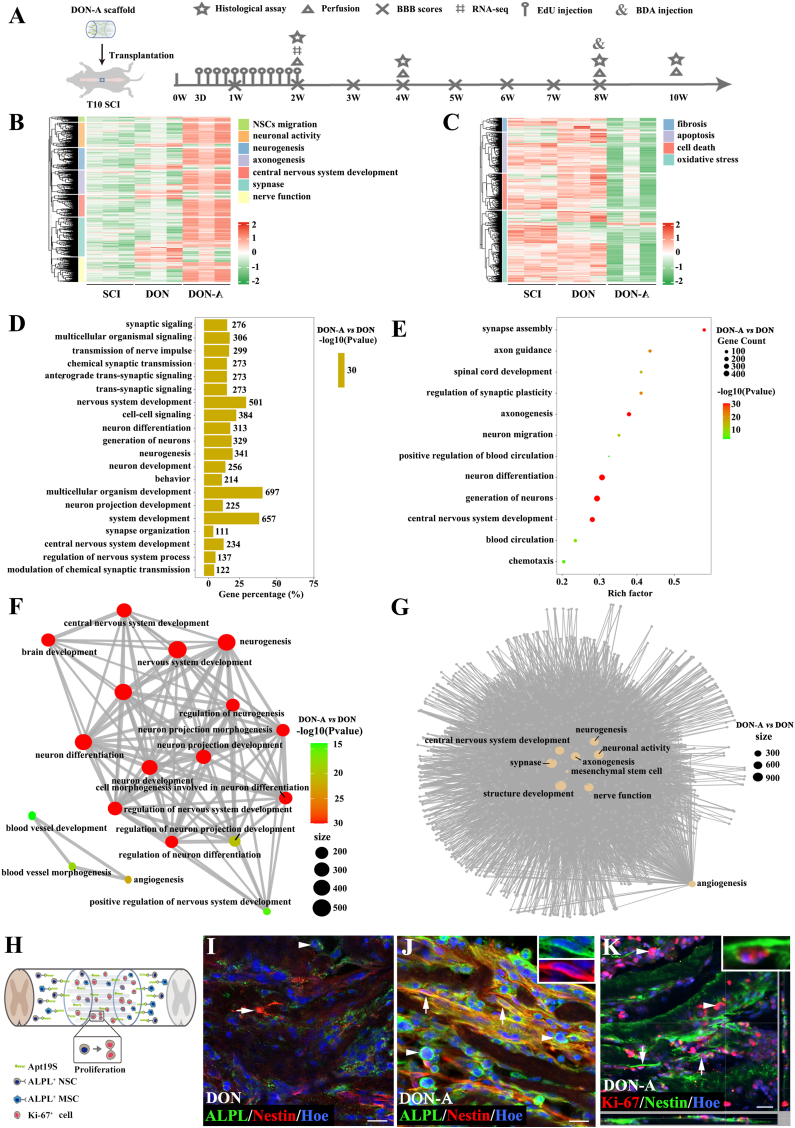
**The DON-A scaffold recruits endogenous NSCs and MSCs to initiate spinal cord repair two weeks after SCI.** (A) Schematic diagram showing the experimental procedures of the current study. (B) Heatmap showing the significantly up-regulated genes related to NSC migration, neuronal activity, neurogenesis, nerve regeneration and synapse formation in the DON-A group. (C) Heatmap showing the significantly down-regulated genes related to fibrosis, apoptosis, cell death and oxidative stress. (D) Bar chart showing the top20 significantly changed terms related to spinal cord repair in GO enrichment analysis in the DON-A group compared to the DON group. (E) Bubble chart showing 12 significant changed GO terms related to NSC migration, neurogenesis, axon regeneration, synaptic formation and reconstruction of blood circulation. (F) GO-GO interaction showing the gene network formed between GO terms related to neurogenesis, central nervous system development and reconstruction of blood circulation. (G) The network diagram showing interactive genes in terms related to the neurogenesis, mesenchymal stem cell and angiogenesis. (H) Schematic diagram showing DON-A group rapidly initiated tissue repair by recruiting endogenous stem cells. (I, J) The expression of Nestin (arrows, NSCs marker protein) and ALPL (arrowheads) on migrated endogenous stem cells in the graft site in the DON (I) and DON-A (J) groups. (K) The expression of Ki-67 (arrowheads, proliferation marker protein) on Nestin^+^ cells (arrows) in the graft site in the DON-A group. Scale bars = 20 μm (I, J); 10 μm (K).

After the *in vitro* observation that DON-A efficiently and specifically recruits NSCs and MSCs and supports their normal differentiation, we transplanted it into the T10 spinal cord of rats with completely transected SCI. We then assessed the tissue repair, gene expression changes, and the behavioral and CMEPs of the rats’ spinal cords at two, four, and eight weeks after the injury, as well as the repair mechanisms involved (Fig. 3A).

Analysis of differential gene expression data from the injury/graft area of the spinal cord two weeks after the injury revealed that the genes that were significantly upregulated in the DON-A group when compared with the DON and SCI groups were enriched for NSCs migration, development of CNS, neurogenesis, neuronal synapse formation, and neural activity (Fig. 3B). These results suggest that in the DON-A group, ALPL-expressing NSCs were recruited, which initiated proliferation, differentiation, axon regeneration, and synapse formation repair, forming a neural development-like microenvironment. Moreover, clustered heatmap analysis of the genes that were differentially expressed in the three groups demonstrated that DON-A markedly suppressed apoptosis, mortality, fibrous scarring, and oxidative stress genes in the injury/graft area (Fig. 3C). Taken together, these data imply that the DON-A group triggered regenerative repair at the two week-time point without ongoing pathological progression and deterioration of the microenvironment. Indeed, the top 20 significant Gene ontology (GO) terms based on the genes that were significantly upregulated in the DON-A vs DON groups were mostly associated with neurodevelopment, neurogenesis, neuronal differentiation, and the formation of neurological functions (Fig. 3D). The significantly enriched GO bubble plots based on upregulated genes suggested that GO terms related to spinal cord development, neuronal development and synaptic formation were also highly enriched in the DON-A group than in the DON group (Fig. 3E). Notably, gene modules associated with blood circulation were also markedly enriched in the DON-A group, implying that the revascularization was initiated by DON-A, along with neural development support (Fig. 3E). The GO–GO network based on GO terms related to spinal repair that were upregulated in the DON-A group than in the DON group revealed strong gene interactions in neurodevelopment and neurodevelopmental regulation, as well as angiogenesis and vascularization (Fig. 3F). A network map of the genes associated with the upregulated GO terms suggested that when compared with the DON group, MSC- and angiogenesis-associated genes in the DON-A group were closely associated with CNS development and the reconstruction of neural tissue structure and function (Fig. 3G). The results of Kyoto Encyclopedia of Genes and Genomes (KEGG) enrichment analysis and protein-protein interaction network analysis indicated that cyclic adenosine monophosphate (cAMP) signaling pathway and Calcium signaling pathway were significantly upregulated in the DON-A group (Fig. S5).

The histological examination of the spinal cord at the two-week time point showed that the longitudinal sections of the spinal cords from the DON-A group had significantly higher levels of Nestin^+^ NSCs migrating into the scaffolds’ interior when compared with the SCI and DON groups (Fig. 3H-K, Fig. S6). These Nestin^+^ cells expressed high ALPL levels, with the results showing that 65 % of the ALPL^+^ cells expressed Nestin in DON-A group (Fig. 3J). We further co-stained ALPL with PAX6 (NSCs marker protein), GFAP, Map2 and MSCs marker (CD73 and CD90) respectively, and confirmed high expression of ALPL on PAX6^+^, CD73^+^ and CD90^+^ cells (Figure S7 and Figure S8), and low or no ALPL expression on GFAP^+^ astrocytes and Map2^+^ neurons (Fig. S7). Additionally, CD68^+^ inflammatory cells and vimentin^+^ myofibroblasts also exhibited low or no ALPL expression (Fig. S8), indicating that Apt19S did not recruit these two cell types, which may exacerbate inflammation and fibrotic scar formation. In contrast, fewer Nestin^+^ cells were observed in the scaffolds in the DON group and the number of the ALPL^+^ cells was also significantly lower than in the DON-A group (Fig. 3I, Fig. S6). Analysis of the two weeks post-injury samples revealed more Ki-67^+^ cells at the center of the DON-A scaffold than in the SCI and DON groups (Fig. 3K, Fig. S9), and 40 % of these Ki-67^+^ cells were Nestin^+^. Double immunofluorescence staining confirmed that DON-A effectively recruited a substantial number of Nestin/SOX2 double-positive NSCs to the injury/transplantation site (Fig. S10). These findings are consistent with the RNA-seq results, indicating that the DON-A group rapidly initiated tissue repair by recruiting endogenous stem cells, specifically endogenous ALPL-rich NSCs. These stem cells exhibited proliferation in the microenvironment of the DON scaffold during the acute phase of SCI, which led to an increase in their level (Fig. 3K, Fig. S9). To determine if as suggested by the RNA-seq data, the Apt19S-recruited NSCs initiate neurodevelopment-like processes for *in situ* neural regeneration and revascularization, the assay time was extended to four and eight weeks for further observation.

### 3.4 NSC differentiation into neurons and oligodendrocytes four weeks after DON-A transplantation

**Fig. 4:**
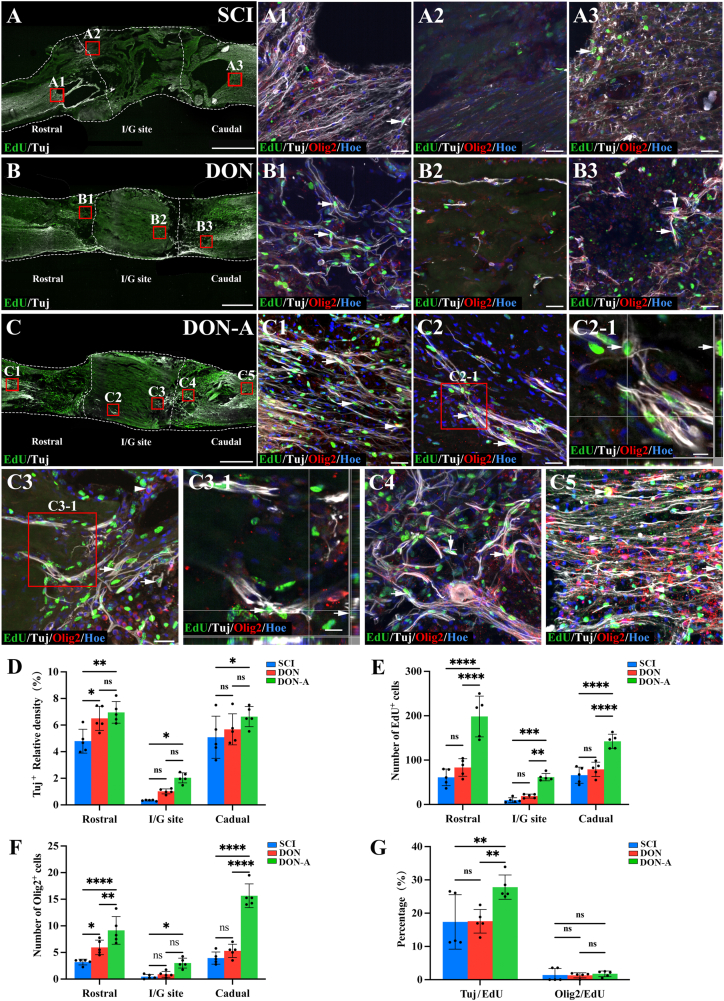
**The differentiation of NSCs towards newborn neurons and oligodendrocytes *in vivo* four weeks after SCI.** (A–C) Low magnification image of sagittal sections of spinal cord showing the expression of Tuj and EdU in the SCI (A), DON (B) and DON-A (C) groups. (A1-A3) Tuj^+^/EdU^+^ cells (arrows) and Olig2^+^/EdU^+^ cells (arrowheads) in the boundary regions rostral to the injury/graft site (A1), at the injury/graft site (A2), and the boundary regions caudal to the injury/graft site (A3) in the SCI group. (B1-B3) Tuj^+^/EdU^+^ cells(arrows) and Olig2^+^/EdU^+^ cells (arrowheads) in the boundary regions rostral to the injury/graft site (B1), at the injury/graft site (B2), and the boundary regions caudal to the injury/graft site (B3) in the DON group. (C1-C5) Tuj^+^/EdU^+^ cells (arrows) and Olig2^+^/EdU^+^ cells (arrowheads) in the regions rostral to the injury/graft site (C1), at the injury/graft site (C2, C3), and in the boundary regions caudal to the injury/graft site (C4) and in the regions caudal to the injury/graft site (C5) in the DON-A group. (C2-1, C3-1) High magnification images showing Tuj^+^/EdU^+^ cells in the middle of graft site. (D) Bar chart showing the quantification of Tuj^+^ nerve fibers in the three groups (*n* = 5, one-way ANOVA with LSD-t post-hoc test, ∗∗*P <* 0.01, ∗*P <* 0.05; ns indicates no significant difference). (E) Bar chart showing the number of EdU^+^ cells in the three groups (*n* = 5, one-way ANOVA with LSD-t post-hoc test, ∗∗∗∗*P <* 0.0001, ∗∗∗*P <* 0.001, ∗∗*P <* 0.01, ns indicates no significant difference). (F) Bar chart showing the number of Olig2^+^ cells in the three groups (*n* = 5, one-way ANOVA with LSD-t post-hoc test, ∗∗∗∗*P <* 0.0001, ∗∗*P <* 0.01, ∗*P <* 0.05; ns indicates no significant difference). (G) Bar chart showing the percentage of Tuj^+^/EdU^+^ and Olig2^+^/EdU^+^ cells among the EdU^+^ cells in the three groups (*n* = 5, one-way ANOVA with LSD-t post-hoc test, ∗∗*P <* 0.01; ns indicates no significant difference). Scale bars = 1 mm (A, B, C); 30 μm (A1-A3, B1-B3, C1-C5); 15 μm (C3-1) and 10 μm (C2-1).

To track cells with the ability to proliferate and differentiate during the acute phase of SCI, we intraperitoneally administered EdU to the rats in each group for 10 days, starting on the third day of the injury. Four weeks post-operation, we examined the boundary and central parts of the injury/graft site of the longitudinal section of the spinal cords in each group. This analysis revealed that when compared with the DON and SCI groups, the DON-A group had a significantly higher number of EdU^+^ cells boundary to the injury/graft site and inside the scaffold (Fig. 4A-E). Although the DON group also had EdU^+^ cells in these areas, their count was significantly lower than in the DON-A group and comparable to the SCI group (Fig. 4A-E). Fluorescence intensity analysis revealed significantly more Tuj^+^ neurites in the rostral regions and at the center of the injury/graft site in the DON-A group when compared with the SCI groups (Fig. 4D). However, Tuj expression at the rostral and caudal end did not differ significantly between the DON-A and DON groups (Fig. 4D). Additionally, in the boundary and central parts of the injury/graft site, Tuj expression was significantly lower in the SCI group when compared with the other two groups (Fig. 4D). The number of Olig2^+^ cells at the boundary parts of the injury/graft site was significantly higher in the DON-A group when compared with the other two groups (Fig. 4F). The DON group had significantly more Olig2^+^ cells in the rostral region when compared with the SCI group but their number did not differ significantly in the injury/graft and in the caudal region (Fig. 4F). Notably, in the DON-A group, a higher proportion of EdU^+^ cells differentiated into Tuj^+^ cells, and numerous EdU^+^/Tuj^+^ neurites grew into the boundary of the scaffold and along the scaffold when compared with the DON and SCI group (Fig. 4C and G). Only a small proportion of EdU^+^ cells differentiated into Olig2^+^ cells and they mainly localized at the rostral and caudal regions of the spinal cord (Fig. 4C and G). These results indicate that the recruited endogenous NSCs exhibited a higher rate of neuronal differentiation in the microenvironment created by DON-A.

### 3.5 Nerve and vascular regeneration four weeks after DON-A transplantation

**Fig. 5:**
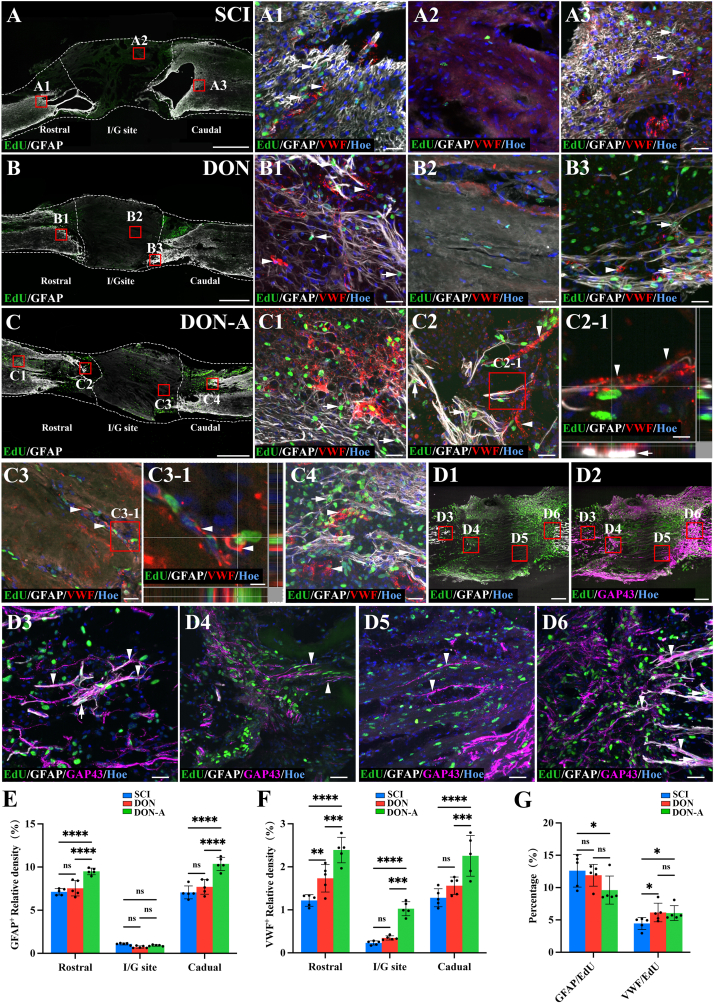
**The newborn astrocytes and vascular endothelial cells *in vivo* four weeks after SCI.** (A–C) Low magnification images of sagittal sections of spinal cord showing the expression of EdU and GFAP in the SCI (A), DON (B) and DON-A (C) groups. (A1-C4) GFAP^+^/EdU^+^ cells and VWF^+^/EdU^+^ cells in the regions rostral to the injury/graft site (A1, B1 and C1), in the boundary regions rostral to the injury/graft site (C2, C2-1), at the injury/graft site (A2, B2, C3, C3-1), and the boundary regions caudal to the injury/graft site (A3, B3 and C4) in the three groups. (D1) Low magnification image of sagittal sections of spinal cord showing the expression of EdU and GFAP in the DON-A groups. (D2) Low magnification image of sagittal sections of spinal cord showing the expression of EdU and GAP43 in the DON-A group. (D3-D6) GFAP^+^/EdU^+^ cells and GAP43^+^ neurites in the regions rostral to the injury/graft site (D3), in the boundary regions rostral to the injury/graft site (D4), at the injury/graft site (D5), and in the boundary regions caudal to the injury/graft site (D6) in the DON-A group. (E, F) Bar chart showing the relative density of GFAP^+^ (E) and VWF^+^ (F)in the three groups (*n* = 5, one-way ANOVA with LSD-t post-hoc test, ∗∗∗∗*P <* 0.0001, ∗∗∗*P <* 0.001, ∗∗*P <* 0.01, ns indicates no significant difference). (I) Bar chart showing the percentage of GFAP^+^/EdU^+^ and VWF^+^/EdU^+^ cells among the EdU^+^ cells in the three groups (*n* = 5, one-way ANOVA with LSD-t post-hoc test, ∗*P <* 0.05, ns indicates no significant difference). Scale bars = 1 mm (A, B, C); 500 μm (D1, D2); 50 μm (D3-D6); 30 μm (A1-A3, B1-B3, C1-C4) and 10 μm (C2-1, C3-1).

Four weeks after the operation, we traced spinal cord endogenous stem cells with EdU to assess their differentiation into astrocytes and vascular endothelial cells. This analysis revealed the presence of GFAP^+^ astrocytes and VWF^+^ endothelial cells at the rostral and caudal regions of the injury/graft site in SCI, DON and DON-A group (Fig. 5A-C). Some GFAP^+^ and VWF^+^ cells were also EdU^+^, indicating that they were recruited during the acute phase of the injury and that they originated from cells capable of dividing and proliferating (Fig. 5A-C). In the SCI group, a small number of GFAP^+^ cells were detected in the center of the injured area (Fig. 5A2). In both DON-A and DON groups, few GFAP^+^ cells were found inside the scaffolds (Fig. 5B2 and 5C3). Most of the GFAP^+^ cells remained in the boundary region of the spinal cord (Fig. 5A-D). In this region, the DON-A group exhibited significantly higher GFAP expression than the DON and SCI groups (Fig. 5E). In the DON-A group, there were significantly more VWF^+^ endothelial cells at the boundary and central regions of the injury/graft site compared to the SCI and DON group (Fig. 5A–C, 5F). Additionally, the graft boundary area of the DON-A group had dense, branched vascularization formed by VWF^+^/EdU^+^ endothelial cells (Fig. 5C2 and 5C4). These blood vessels could regenerate along the channel of the DON scaffold and into the central region of the graft site, suggesting that the endogenous stem cells recruited by DON-A had good angiogenic potential (Fig. 5C3, 5C3-1). In the graft boundary area, the endfeet-like structures formed by the EdU^+^/GFAP^+^ astrocytes included the neovascularization formed by the differentiation of EdU^+^/VWF^+^ cells (Fig. 5C2-1). This suggests that these astrocytes may be involved in the establishment of a blood–spinal cord barrier. Analysis of the differentiation rate of the EdU^+^ cells indicated that the proportion of cells differentiating into GFAP^+^ astrocytes was significantly higher in the SCI groups than in the DON-A group (Fig. 5G). However, the rate of differentiation into Tuj^+^ neurons was significantly lower in the DON and SCI groups (Fig. 4G). These results suggest that the high ALPL-expressing NSCs had better neuronal differentiation potential. Additionally, the proportion of EdU^+^ cells that differentiated into VWF^+^ endothelial cells was higher in the DON-A and DON group than in the SCI group (Fig. 5F). Taken together with the results from the RNA-seq analysis after two weeks, these results indicate that the ALPL^+^ MSCs recruited by Apt19S may have more angiogenic potential and might improve the microenvironment to promote angiogenesis.

To investigate the relationship between the increased number of GFAP^+^/EdU^+^ cells and nerve regeneration in the DON-A group, we analyzed the expression of GFAP and the growth-associated protein, GAP43, which is highly expressed in the axon of neurons with high regenerative ability. At the rostral and caudal regions in the transplantation area of the DON-A group, the neurites of GFAP^+^/EdU^+^ astrocytes tended to grow parallel to the longitudinal axis of the spinal cord and were accompanied by GAP43 positive (GAP43^+^) neurites, without dense glial scarring to block the growth of GAP43^+^ neurites (Fig. 5D1-5D6). Instead, the GFAP^+^/EdU^+^ astrocytes guided the regeneration of the GAP43^+^ neurites into the boundary region of the injury/graft site and inside the DON-A scaffold (Fig. 5D3 and 5D6).Interestingly, GAP43^+^ neurites regenerated into the center of the scaffold and did not remain at the boundary region of the injury/graft site as astrocytes, and many GAP43^+^ neurites grew straight, along the scaffold's channel (Fig. 5D4 and 5D5).

### 3.6 Recovery of motor function and nerve regeneration

**Fig. 6:**
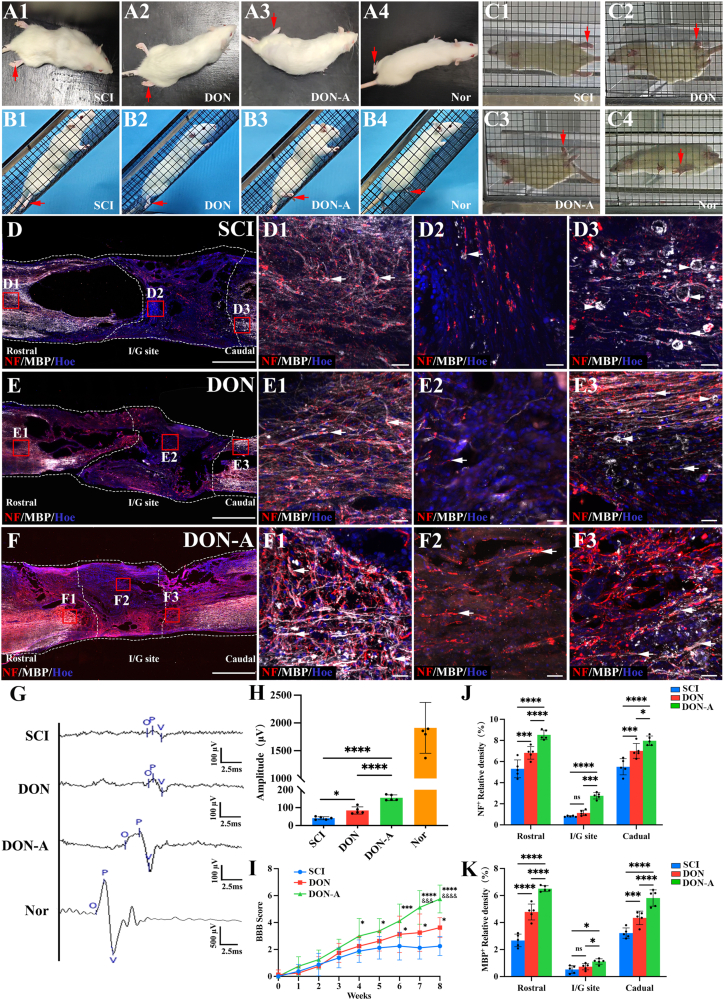
**Motor function recovery, electrophysiological improvement and nerve fiber regeneration eight weeks after SCI.** (A1-A4) Open-field observation. (B1-B4) Inclined grid climbing test. (C1-C4) Horizontal ladder walking tests. (D–F) Low magnification images of sagittal sections of spinal cord showing the expression of NF and MBP in the SCI (D), DON (E) and DON-A (F) groups. (D1-F3) The regenerated NF^+^ nerve fibers wrapped with MBP^+^ cells in the regions rostral to the injury/graft site (D1, E1, F1, arrows), at the injury/graft site (D2, E2, F2, arrows) and in the regions caudal to the injury/graft site (D3, E3, F3, arrows) in the three groups. Myelin sheath collapse was observed (D3, E3 arrowheads) (G) CMEPs were detected via a TMS instrument. (H) Bar chart showing that the CMEP amplitudes of rats in the four groups (*n* = 5, one-way ANOVA with LSD-t post-hoc test, ∗∗∗∗*P <* 0.0001, ∗*P <* 0.05). (I) BBB scores for assessing locomotor recovery of rats in the four groups (*n* = 8, one-way ANOVA with LSD-t post-hoc test at each time point, ∗ and & indicate statistical significance when DON-A compared with the SCI and DON groups, respectively, ∗∗∗∗*P <* 0.0001, ∗∗∗*P <* 0.001, ∗*P <* 0.05 ^&&&&^*P <* 0.0001, ^&&&^*P <* 0.001). (J, K) Bar chart showing the relative density of NF^+^ and MBP^+^ cells in the three groups (*n* = 5, one-way ANOVA with LSD-t post-hoc test, ∗∗∗∗*P <* 0.0001, ∗∗∗*P <* 0.001, ∗*P <* 0.05, ns indicates no significant difference). Scale bars = 1 mm (D, E, F); 30 μm (D1-D3, E1-E3, F1-F3).

After SCI surgery, we conducted weekly behavioral tests to evaluate the rats’ motor function recovery and performed CMEP and nerve regeneration detection after eight weeks (Fig. 6A-K). Rats did not display hindlimb spasticity or sensory abnormalities such as leg biting during eight weeks of observation. The results showed that after four weeks, the DON-A group exhibited significantly higher joint movement when compared with the SCI and DON groups and the movement continued to increase, reaching a BBB score about six in the eight week, showing extensive movement in two joints and slight movement in the third joint (Fig. 6A1-6A4, 6I,Video S1). In the DON-A group, significant improvements in hindlimb function was also observed in the inclined-grid climbing test and the horizontal ladder walking tests which allowed for a more detailed observation of the voluntary movements than the open-field locomotor test (Fig. 6B3, 6C3,Video S2 and S3). After eight weeks of SCI, there was no placement reflex in the SCI and DON groups. When the forelimbs climbed the diagonal grid, the hindlimbs were dragged behind and often fell (Fig. 6B1-6B2, 6C1-6C2,Video S2 and S3). In contrast, rats in the DON-A group generally showed pronounced placement reflexes in their hindlimbs, and their hind feet would occasionally tread on the grid (Fig. 6B3, 6C3,Video S2 and S 3).

Next, histological analysis was used to determine whether the motor recovery of the rats was accompanied by nerve regeneration. We examined the longitudinally-sectioned spinal cord samples (Fig. 6D-F). Notable neurofilament positive (NF^+^) nerve fiber and myelin sheath collapse was observed in the caudal regions of the injury area in the SCI and DON group (arrowheads in Fig. 6D3 and 6E3). The DON-A group exhibited significantly higher levels of NF^+^ nerve fiber in the injured/grafted area and the rostral and caudal region, and that the NF^+^ nerve fibers in the rostral and caudal region were surrounded by myelin basic protein positive (MBP^+^) myelin sheaths without myelin collapse (Fig. 6F). Moreover, at the injury/graft site, the scaffold gradually degraded and was replaced by regenerated neural tissue, more NF^+^ nerve fibers without MBP encapsulation were found to regenerate straight into the scaffold in the DON-A group than in the SCI and DON group (Fig. 6F2). Furthermore, the relative NF and MBP fluorescence intensities were significantly higher in the DON-A group than in the DON and SCI groups (Fig. 6J and K). The expression levels of NF and MBP were also significantly higher in the rostral and caudal regions of the spinal cords of the DON group when compared with the SCI group (Fig. 6J and K). However, no significant differences were observed in the central region of the injured area between DON and SCI group (Fig. 6J and K). Consistent with the results of the nerve regeneration, the CMEP results showed that the DON-A group had a significantly higher action potential amplitude upon stimulation of the motor cortex when compared with the SCI and DON groups (Fig. 6G and H).

### 3.7 Immune and regenerative microenvironment after DON-A transplantation

**Fig. 7:**
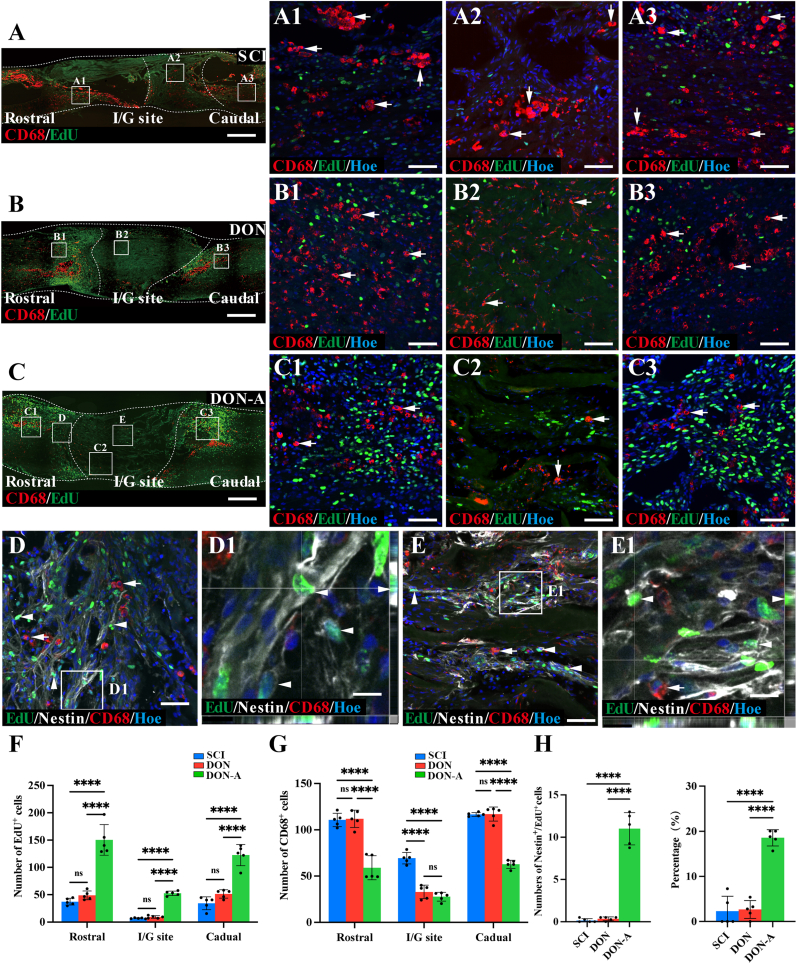
**The inflammatory and regenerative microenvironment *in vivo* eight weeks after SCI.** (A–C) Low magnification image of sagittal sections of spinal cord showing the expression of EdU and CD68 in the SCI (A), DON (B) and DON-A (C) groups. (A1-C3) CD68^+^/EdU^+^ cells in the boundary regions rostral to the injury/graft site (A1, B1, C1, arrows), at the injury/graft site (A2, B2, C2, arrows), and the boundary regions caudal to the injury/graft site (A3, B3, C3, arrows) in the three groups. (D) Nestin^+^/EdU^+^ cells and CD68^+^ cells in the regions rostral to the injury/graft site in the DON-A group (arrowheads). (E) Nestin^+^/EdU^+^ cells and CD68^+^ cells at the injury/graft site in the DON-A group (arrowheads). (F, G) Bar chart showing the number of EdU^+^ and CD68^+^ cells in the regions rostral/caudal to the injury/graft site and at the injury/graft site in the three groups (*n* = 5, one-way ANOVA with LSD-t post-hoc test, ∗∗∗∗*P <* 0.0001, ns indicates no significant difference). (H) Bar chart showing the number of Nestin^+^/EdU^+^ cells at the injury/graft site in the three groups. And the bar chart showing the percentage of Nestin^+^/EdU^+^ cells among the EdU^+^ cells in the three groups (*n* = 5, one-way ANOVA with LSD-t post-hoc test, ∗∗∗∗*P <* 0.0001, ns indicates no significant difference). Scale bars = 500 μm (A–C); 50 μm (A1-A3, B1-B3, C1-C3, D, E); 10 μm (D1, E1).

To investigate the immune and regenerative microenvironment associated with motor function recovery, we examined the distribution of EdU^+^ and CD68^+^ cells in longitudinally-sectioned spinal cord samples eight weeks after the operation. This analysis revealed that the number of EdU^+^ cells in the central and boundary regions of the injury/graft site was significantly higher in the DON-A group than in the DON and SCI groups (Fig. 7A-F). Additionally, the number of EdU^+^ cells at four weeks and eight weeks were not significantly decreased (Fig. S11). In contrast, the number of CD68^+^ cells in the rostral and caudal boundary regions of the injury/graft site was significantly lower in the DON-A group when compared with the DON and SCI groups (Fig. 7A-F). However, the levels of CD68^+^ inflammatory cells in the center of the injury/graft site did not differ significantly between the DON-A and DON groups, although for both groups, they were significantly lower than in the SCI group (Fig. 7A2-7C2). In the central, rostral, and caudal boundary regions of the injury/graft site, the SCI group had higher levels of CD68^+^ cells, which had larger cell bodies containing abundant cytoplasmic particles (Fig. 7A). The CD68^+^ cells in the DON-A group were more dispersed and smaller than in the SCI and DON groups, with elongated CD68^+^ cells in the boundary and central regions, which closely resembled normal spinal cord microglia (Fig. 7C). None of the groups exhibited EdU/CD68 double-positive cells. These results indicate that the endogenous stem cells recruited by DON-A may contribute to the improvement of the spinal cord's immune microenvironment.

In the SCI and DON group, eight weeks post-surgery, a small number of EdU^+^ and Nestin^+^ cells were observed in the rostral and caudal regions to the injury/graft site (Fig. S12). However, no Nestin^+^ cells were detected in the injury/graft site in the SCI and DON groups (Fig. S12). In contrast, the DON-A group had groups of Nestin^+^ cells in the central and boundary areas of the scaffolds and some of them were EdU^+^ (Fig. 7D-E), with a much higher proportion of EdU^+^ cells being Nestin^+^ than in the SCI and DON groups (Fig. 7H). These findings suggest that the endogenous stem cells recruited by DON-A significantly reduced the activation and aggregation of CD68^+^ cells, leading them towards a more normal immune cell morphology, instead of an activated state. Additionally, the endogenous stem cells colonized the inside of the scaffolds and created a microenvironment that closely resembled normal spinal cord tissue. DON-A transplantation can not only promote neural stem cell differentiation into neurons but also allow some NSCs to maintain their stemness in this microenvironment, enabling the spinal cord to maintain their repair potential.

### 3.8 The formation of endogenous neurons relays

**Fig. 8:**
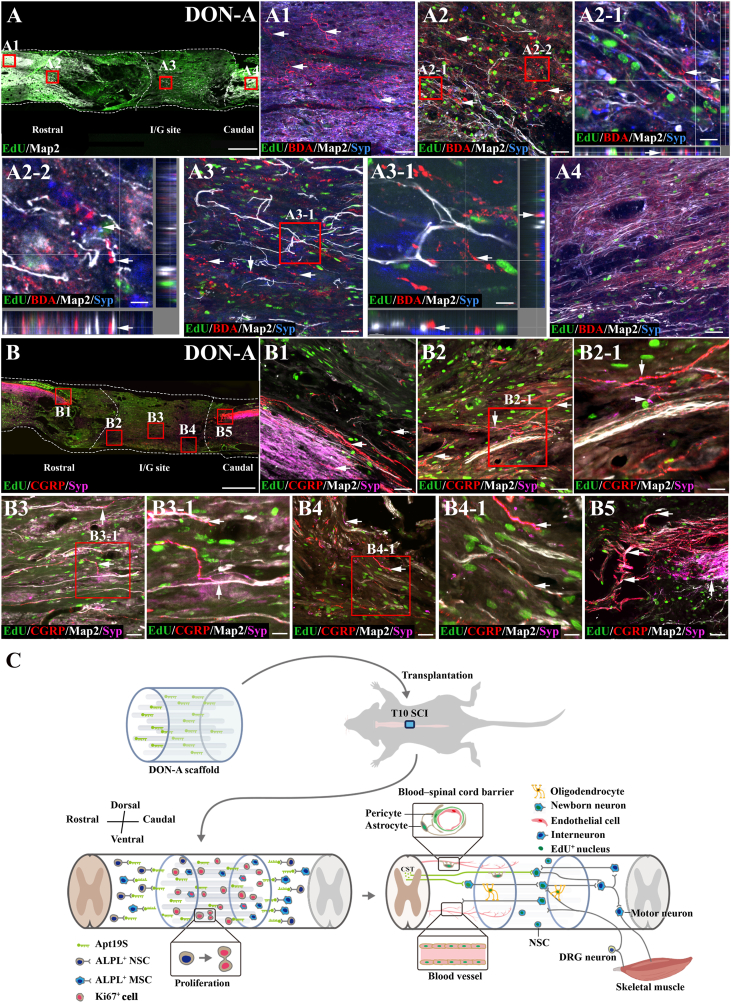
**The regeneration of CST and CGRP** + **nerve fibers, and their connections with newborn neurons.** (A) Low magnification image of sagittal sections of spinal cord showing the expression of EdU and Map2 in the DON-A group. (A1-A4) The BDA^+^ CST regenerating to the regions rostral to the injury/graft site (A1, arrows), the boundary regions rostral to the injury/graft site (A2, arrows), the injury/graft site (A3, arrows), but not to the regions caudal to the injury/graft site (A4) in the DON-A group. (A2-1, A3-1) The regenerative BDA^+^ CST formed synaptic connection with Map2^+^/EdU^+^ cells in the boundary regions rostral to the injury/graft site (A2-1, A2-2, arrows) and at the injury/graft site (A3-1, arrows). (B) Low magnification image of sagittal sections of spinal cord showing the expression of EdU, Map2, CGRP and Syp in the DON-A group. (B1-B5) The regeneration of CGRP^+^ nerve fibers in the boundary regions rostral to the injury/graft site (B1, arrows), at the injury/graft site (B2, B3, B4, arrows), and in the boundary regions caudal to the injury/graft site (B5, arrows). (B2-1, B3-1, B4-1) The regenerative CGRP^+^ nerve fibers formed synaptic connections with Map2^+^/EdU^+^ cells at the injury/graft site. (C) Schematic diagram showing the underlying mechanism of spinal cord repair via DON-A scaffold. Scale bars = 1 mm (A, B); 50 μm (A1-A5, B1-B5); 20 μm (A2-1, B2-1, B3-1, B4-1); 10 μm (A2-2, A3-1).

Eight weeks post-surgery, many Map2^+^/EdU^+^ newborn neurons were detected inside the scaffolds in the DON-A group (Fig. 8A). To determine if these newborn neurons functionally integrated with the regenerated nerve fibers throughout the spinal cord to reconstruct the neural pathway, we injected BDA into the somatosensory-motor cortex and tracked the integration of CST with the newborn neurons. The CST nerve fibers regenerated in the rostral end of the DON-A grafted area and inside the scaffolds (Fig. 8A1-8A3). A close relationship was observed between CST nerve fibers and Map2^+^/EdU^+^ newborn neurons, accompanied by the expression of the synaptic marker protein, Syp, at the contact points, indicating that CST nerve fibers formed synaptic connections with newborn neurons (Fig. 8A2 and 8A3). However, no CST^+^ nerve fibers were detected at the caudal end of the injury/graft site (Fig. 8A4). Additionally, the integration of calcitonin gene related peptide positive (CGRP^+^) sensory ascending nerve fibers with newborn neurons revealed CGRP^+^ nerve fiber regeneration in the boundary and central regions of the injury/graft site (Fig. 8B, B1-8B5). These CGRP^+^ nerve fibers were closely contacted with Map2 and EdU double-positive neurons and Syp were observed at CGRP + nerve fiber (Fig. 8B2-8B4). These results indicated that newborn neurons in the DON-A scaffolds could establish synapses with regenerated ascending and descending spinal cord nerve fibers, achieving functional integration and reconstructing neural pathways (Fig. 8C). In a rat that underwent DON-A transplantation and was sacrificed at week 16, EdU-positive mature neurons co-expressing NeuN and NF were still detectable. These neurons were localized within the undegraded linear scaffold of the DON (Fig. S13).

## 4 Discussion

In adult mammals, tissue regeneration and structural repair are crucial for neural function reconstruction after CNS injury [6,21,27]. Stem cells are central to the regeneration of injured spinal cords but the absence of endogenous stem cells in severely damaged spinal cords and their poor survival in the hostile injured microenvironment, remain major obstacles to achieving *in situ* regeneration and functional reconstruction [2,28,29]. To address these challenges, we innovatively loaded Apt19S, which specifically binds to ALPL on the membranes of stem cells [17,19], into DON (DON-A). The DON-A scaffold can support the regenerative channels of the spinal cord and slowly release Apt19S, while specifically recruiting ALPL^+^ NSCs and MSCs, effectively promotes *in situ* nerve and vascular regeneration, as well as functional reconstruction of the neural pathways in injured spinal cord.

### 4.1 DON-A efficiently recruits NSCs and MSCs and supports their differentiation *in vitro*

NSCs can differentiate into neurons, astrocytes, and oligodendrocytes, which are vital for neural repair. Previous research has mainly focused on transplanting exogenous NSCs or activating endogenous NSCs for SCI repair [2,8,30]. It is confirmed that transplanted MSCs protect injured nerves and promote nerve regeneration through paracrine and immunomodulatory effects [4,31]. However, few studies have investigated the concurrent activation and recruitment of endogenous NSCs and MSCs for SCI treatment. Here, we designed the DON-A scaffold, which can stabilize and persistently release Apt19S, by exploiting Apt19S's ability to specifically bind to ALPL on the stem cell membranes [16,17]. Furthermore, the Apt19S gradient formed by DON-A significantly attracted and promoted the migration of NSCs and MSCs. Importantly, the DON-A scaffold did not alter the normal phenotypic differentiation of NSCs and it favor MSC differentiation into vascular endothelial cells, which are essential for nerve regeneration and *in situ* revascularization of the injured spinal cord. Based on these findings, we transplanted DON-A into the injured spinal cords to assess whether it could simultaneously recruit endogenous NSCs and MSCs to promote *in situ* spinal cord regeneration.

### 4.2 The DON-A transplant efficiently initiated tissue regeneration and repair

Inflammatory cytokines are known to activate dormant spinal cord NSCs during the acute phase of SCI [2,7]. However, sustained inflammation, hypoxia–ischemia, and oxidative stress in this microenvironment often lead to NSCs apoptosis, ultimately depleting their quantity [8,10]. Reversing the unfavorable microenvironment during the acute phase of SCI is key to the initiation of tissue regeneration and repair [4,25,32].

The application of neurotrophic factors, such as neurotrophin-3, glial cell line-derived neurotrophic factor, and drugs like metformin may improve the injured microenvironment by inhibiting endogenous NSC apoptosis and promoting their differentiation to engage in regeneration and repair [[33], [34], [35]]. Using SDF-1 to recruit endogenous NSCs along with neuroprotective and differentiation-inducing factors can promote the differentiation of more NSCs into neurons and promote neural pathway reconstruction [3,32]. These studies suggest that successful regeneration and repair depend on the number of endogenous NSCs that migrate to the injury, their survival rate, and their committed differentiation. However, in addition to endogenous NSCs, SDF-1 also attracts other cell types, including macrophages and fibroblasts [36,37]. Hence, there is a need for drugs that can efficiently and specifically recruit endogenous stem cells. We hypothesized that the recruitment of endogenous stem cells with high ALPL expression levels to the SCI using Apt19S might achieve better repair effects in the CNS-specific niche created by DON [17,18,21]. Indeed, our results show that Apt19S specifically recruits ALPL-rich NSCs and MSCs. Because macrophages and myofibroblasts express very low ALPL, the DON-A transplantation was associated with a lower risk of inflammation and fibrous scarring [38].

RNA-Seq analysis two weeks after DON-A transplantation confirmed a significant enrichment for GO terms related to NSCs migration, CNS development, axonogenesis, and nerve function in the DON-A group when compared with the DON and the SCI groups, whereas GO terms associated with apoptosis, cell death, fibrosis, and oxidative stress were significantly downregulated. The top GO percentage barplot and the GO enrichment bubble diagram indicated that DON-A transplantation rapidly reversed the hostile microenvironment for nerve regeneration by recruiting many endogenous stem cells in the acute phase of the SCI, thereby forming a regenerative state that resembles the developing spinal cord. GO–GO interaction analysis suggested that MSC recruitment was vital for rapid regeneration initiation. KEGG enrichment analysis and protein-protein interaction network analysis indicated that cAMP signaling pathway and Calcium signaling pathway may be the key signaling pathway in which DON-A plays a key modulation role in cell function.

Histological analysis in the second week confirmed the significant presence of NSCs and MSCs in the DON-A graft site. Significantly, a considerable amount of Ki-67^+^ cells were present in the scaffold, including some that were Nestin^+^. Apt19S likely recruited ALPL^+^ stem cells with a heightened proliferation and differentiation potential [17,18], enabling them to continue proliferating in the regenerative microenvironment created by DON [21]. This contributed to an increase in the amount of endogenous stem cells, which was critical for the rapid reversal of the unfavorable microenvironment and the *in situ* regeneration of spinal cord tissues.

### 4.3 DON-A transplantation achieved *in situ* nerve and vascular regeneration

The key challenge for *in situ* spinal cord regeneration is how to replace pathological cavities and scars while constructing regenerative channels [21,22,39]. However, numerous studies have indicated that neither stem cell transplantation nor endogenous stem cell recruitment can achieve tissue repair without sufficient angiogenesis [11,[39], [40], [41]]. Crucially, DON-A has uniformly distributed longitudinal channels and small pores on the channel wall, which has structural advantages for the construction of regenerative channels and the recruitment of endogenous NSCs and MSCs to drive nerve and vascular regeneration.

In this study, proliferating cells were labeled with EdU during the acute injury phase, and histological analyses four weeks after DON-A transplantation confirmed a significant increase in EdU^+^ cells in the boundary and central areas of the graft when compared with the SCI and DON groups. This observation was consistent with RNA-seq and histology results obtained after two weeks. The EdU^+^ cells included endogenous stem cells recruited by Apt19S and proliferating stem cells, which could differentiate into neurons, astrocytes, and oligodendrocytes, and Tuj^+^ neurons were the most abundant. Many EdU^+^/Tuj^+^ cells grew into the boundary area of the DON-A scaffold and straight along the scaffold. The number of EdU^+^/Tuj^+^ cells was lower in the DON and SCI groups when compared with the DON-A group. Nevertheless, both DON and SCI groups exhibited significantly higher GFAP^+^ cells than the DON-A group, probably because of limited proliferation and neuronal differentiation of endogenous NSCs in the injured microenvironment [13,32]. In contrast, the ALPL^+^ endogenous NSCs recruited by Apt19S had greater potential to migrate, proliferate, and differentiate into neurons, which was more beneficial for rapid spinal cord regeneration and repair.

Promoting angiogenesis is another key advantage of DON-A. Four weeks after DON-A transplantation, the number of neovessels correlated positively with the amount of EdU^+^ cells. When compared with the SCI and DON groups, the DON-A group had a higher proportion of EdU^+^ cells that differentiated into VWF^+^ cells, which significantly enhanced angiogenesis at the boundary area and inside the scaffold. Histological and RNA-seq analyses showed that the endogenous MSCs recruited by Apt19S played a crucial role in reversing the unfavorable microenvironment and promoting differentiation into endothelial cells for neovascularization. Revascularization is also essential for the survival, proliferation, and differentiation of endogenous NSCs [11,13,39]. Interestingly, in the DON-A group, EdU^+^ astrocytes formed endfeet-like structures that encircled the neovascularization formed by EdU^+^ endothelial cells. These results suggest the synergy between endogenous NSCs and MSCs makes DON-A transplantation highly valuable for *in situ* SCI repair.

The traditional view is that after SCI, reactive and proliferating astrocytes play an important role in the formation of glial scars and inflammatory cytokines secretion, which impedes nerve regeneration [14,42,43]. However, our evidence showed that in the DON-A group, EdU^+^ astrocytes effectively guided GAP43^+^ nerve fiber regeneration and which indicated that ALPL^+^ NSC-derived naive astrocytes played a vital role in promoting and guiding axonal regeneration.

### 4.4 Endogenous neuronal relays reconstructed the neural pathway

Prolonging the study to eight weeks revealed further improvement in the rats’ hind limb motor function and spinal cord electrophysiological conduction in the DON-A group. Consistent with this improvement, a significant increase in the amount of EdU^+^ cells at the injury/graft site, as well as more regenerated axon and myelin sheaths were detected. Moreover, the results demonstrated that the DON-A scaffold gradually degraded and was replaced by regenerated neural tissue. There was successful tissue integration at the boundary area of the spinal cord and the DON-A graft. However, cavities were still obvious at the injury/graft boundary area in the DON group, while the SCI group presented with scar tissue and cavities in the injured area, accompanied by severe myelin degeneration at the rostral and caudal sites to the lesion. Additionally, the injury/graft site of the SCI and DON groups exhibited a significantly higher accumulation of CD68^+^ cells than in the DON-A group. These findings indicate that the SCI and DON groups, which lacked endogenous stem cells, progressed to the chronic injury phase and experienced continued neurodegeneration [43,44]. Notably, in the DON-A group, a proportion of EdU^+^ cells in the injury/graft site retained Nestin expression. This finding suggests that the DON-A might markedly restored a microenvironment that resembled the normal spinal cord, thereby enabling the endogenous NSCs to maintain asymmetric division for self-renewal [9,10]. The above results indicated that the simultaneous recruitment of NSCs and MSCs may help reduce the activation of CD68^+^ cells, thereby maintaining microenvironment homeostasis and preventing secondary injuries [4].

When compared with other immunoregulatory strategies for protecting and promoting nerve regeneration [2,45,46], DON-A transplantation recruited endogenous stem cells to counter inflammation, thereby ensuring the endogenous stem cells survival, proliferation, and differentiation. It also facilitated the differentiation of endogenous NSCs into neurons, thereby establishing a pathway for neural information transmission. Moreover, the recruitment of endogenous stem cells presented no risk of immune rejection or the need for immunosuppressants. It is certain that, the endogenous newborn neurons form synapses with the regenerating nerve fibers and achieve functional integration more easily than transplanted neurons [34,35]. Analysis of the descending CST and the ascending CGRP nerve fibers confirmed that the newborn neurons could establish synaptic connections with the motor and sensory nerve fibers, which was essential for the recovery of motor and sensory functions following the transected SCI [47,48].

### 4.5 Strengths and limitations of the study

In the rats with the transplantation of DON-A after the removal of 2 mm spinal cord tissue, the average BBB score at the eight weeks was about 6. Although the score was significantly increased compared with that of the control group, it was still relatively lower than that in the previous study on transplanting pre-differentiated neurons. However, in the curve of the BBB score, it can be observed that the DON-A group showed a linear increase from four to eight weeks, suggesting that the BBB score may further improve with longer observation time. We speculate that synapse formation and functional maturation take time, as Apt19S mainly recruits endogenous stem cells and allows for their transient proliferation in the first four weeks post-injury and newborn neurons gradually develop and mature to form synapses with regenerating nerve fibers after the 4th weeks. We also observed numerous Nestin^+^ cells in the transplanted site of the DON-A group at eight weeks, which suggests that combining DON-A transplantation with neurotrophic factors may promote neuronal differentiation and maturation [34,35]. Furthermore, the additional neural modulation techniques such as spinal epidural electrical stimulation are also potential to increase the number of newborn neurons in the injury site and promote synapse maturation [30,44,48]. These strategies may allow the BBB score to reach or even surpass 8 points, and we will further explore the therapeutic effect of these strategies in the future. Additionally, as RNA-seq can only detect gene expression changes at the tissue level but not in individual cells in current studies, single-cell RNA-Seq could be further employed to identify the subgroups of NSCs and MSCs that are crucial for neurogenesis and revascularization in the DON-A group, and to find out the further mechanism and interpret the key cell types and critical regulatory genes involved *in situ* spinal cord regeneration.

The clinical advantages of DON-A are as follows: First, DON is sourced from porcine optic nerve tissue, facilitating standardized preparation and mass production. Following decellularization, it exhibits excellent immunocompatibility and does not induce immune rejection in rats, as demonstrated in previous studies [21,22]. Porcine-derived decellularized biomaterials, such as pericardium and bone, have been extensively utilized in clinical settings and exhibit long-term stability at the transplantation site [49]. At 16 weeks post-transplantation, the DON-A scaffold was seamlessly integrated with spinal cord tissue and maintained channels conducive to axonal regeneration, confirming its long-term stability and structural support for the spinal cord. Second, oligonucleotide aptamer drugs, including apt19s, are widely adopted in clinical practice due to their high safety profile and acceptance by clinicians [50,51]. Consequently, DON-A demonstrates significant potential for repairing central nervous system injuries.

## 5 Conclusion

To address the challenge of SCI repair, we designed the DON-A scaffold, which efficiently recruited endogenous NSCs and MSCs via sustained release of Apt19S that specifically binds to the ALPL receptor, thereby rapidly initiating repair in the “incubation” microenvironment provided by the scaffold. This approach facilitated *in situ* nerve and vascular regeneration in the injured spinal cord, forming endogenous neuronal relays that reconstructed both the ascending and descending neural pathways. Our study challenged the traditional viewpoint that endogenous stem cells are inadequate to support self-repair after SCI while overcoming the clinical transformation limitations associated with cell transplantation [8,30,52]. It presents a novel strategy for simultaneously recruiting endogenous NSCs and MSCs to drive *in situ* spinal cord repair.

## CRediT authorship contribution statement

**Bi-Qin Lai:** Writing – review & editing, Writing – original draft, Funding acquisition, Data curation, Conceptualization. **Rong-Jie Wu:** Writing – original draft, Formal analysis, Data curation, Conceptualization. **Chuang-Ran Wu:** Formal analysis, Data curation. **Hai-Yang Yu:** Data curation. **Jing Xu:** Data curation. **Shang-Bin Yang:** Data curation. **Zheng-Hong Chen:** Data curation. **Xing Li:** Data curation. **Yi-Nan Guo:** Data curation. **Yue Yang:** Data curation. **Ming-Tian Che:** Data curation. **Ting-Ting Wu:** Data curation. **Guang-Tao Fu:** Data curation. **Yu-Hui Yang:** Data curation. **Zhen Chen:** Data curation. **Nan Hua:** Data curation. **Rui Liu:** Funding acquisition, Data curation. **Qiu-Jian Zheng:** Funding acquisition, Data curation, Conceptualization. **Yuan-Feng Chen:** Writing – review & editing, Funding acquisition, Data curation.

## Declaration of competing interest

The authors declare that they have no known competing financial interests or personal relationships that could have appeared to influence the work reported in this paper.

## Data Availability

Data will be made available on request.
