# Supplementary material for: DON-Apt19S bioactive scaffold transplantation promotes in situ spinal cord repair in rats with transected spinal cord injury by effectively recruiting endogenous neural stem cells and mesenchymal stem cells
Source: Mater Today Bio. 2025 Apr 10;32:101753. doi: 10.1016/j.mtbio.2025.101753 (PMC12019207; doi:10.1016/j.mtbio.2025.101753)
Supplement: Multimedia component 1 [file mmc1.docx]

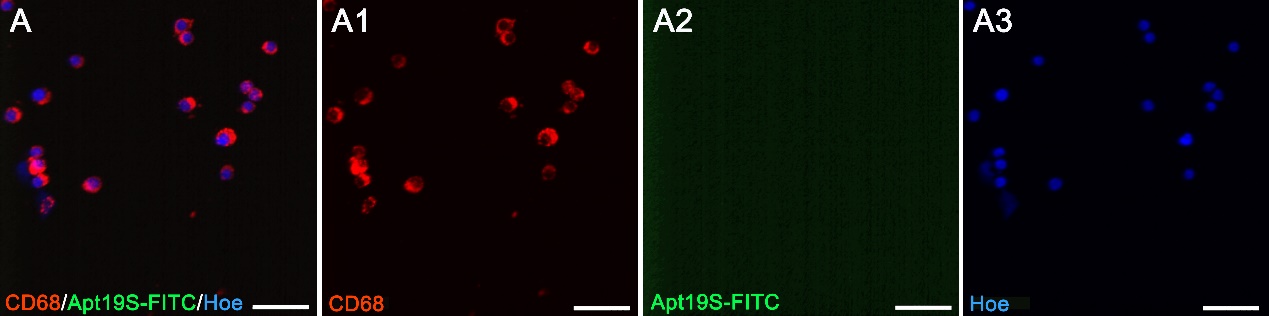


**Figure S1 FITC-Apt19S could not be uptaken by macrophages.** (A) CD68^+^ macrophage (A1, A3) were not labeled by FITC-Apt19S (A2) during *in vitro* co-incubation. Scale bars = 20 μm (A, A1-A3).


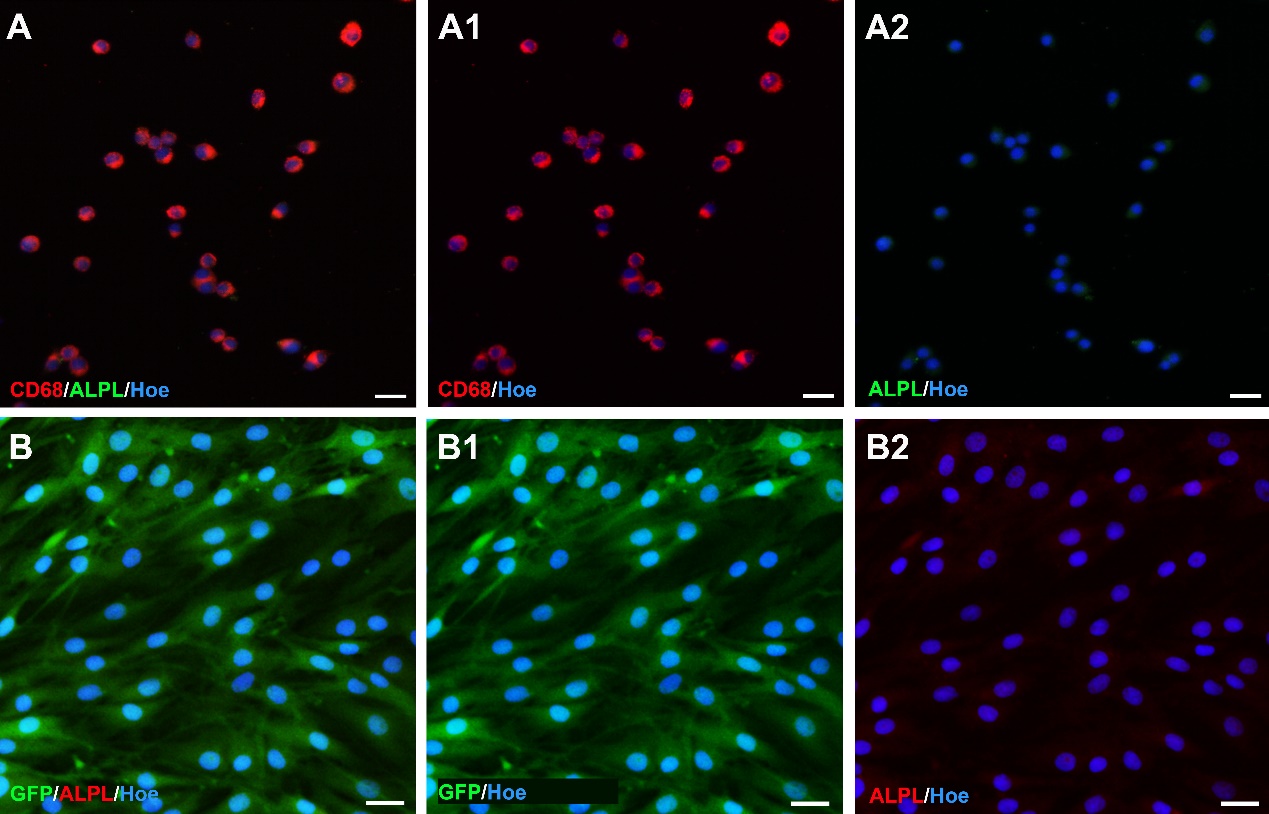


**Figure S2 The expression of ALPL on macrophages and vascular smooth muscle cells *in vitro*.** (A) CD68^+^ macrophage showing very low expression of ALPL. (B) Vascular smooth muscle cells with GFP as report gene also showing very low expression of ALPL. Scale bars = 20 μm (A,B).


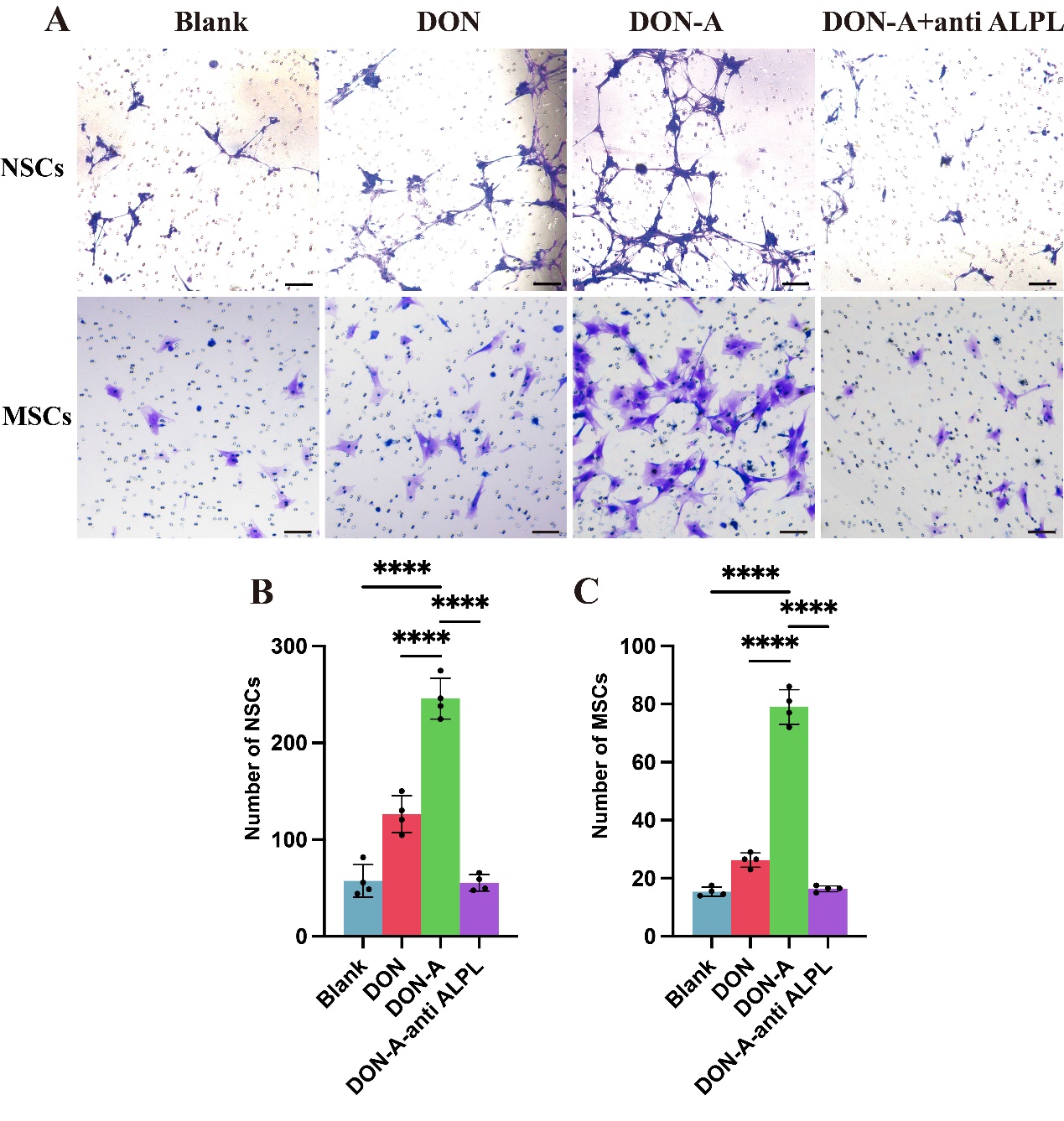


**Figure S3 ALPL antibody abolished enhanced migration of NSCs and MSCs via Apt19S.** (A) Crystal violet staining of migrated NSCs and MSCs in the Blank, DON, DON-A and DON-A+anti-ALPL group in a Transwell system. (B-C) Bar chart showing the number of migrated NSCs (B) and MSCs (C) per high power filed (*n*=4, Student’s t-tests, ****P < 0.0001). Scale bars = 100 μm in (A).


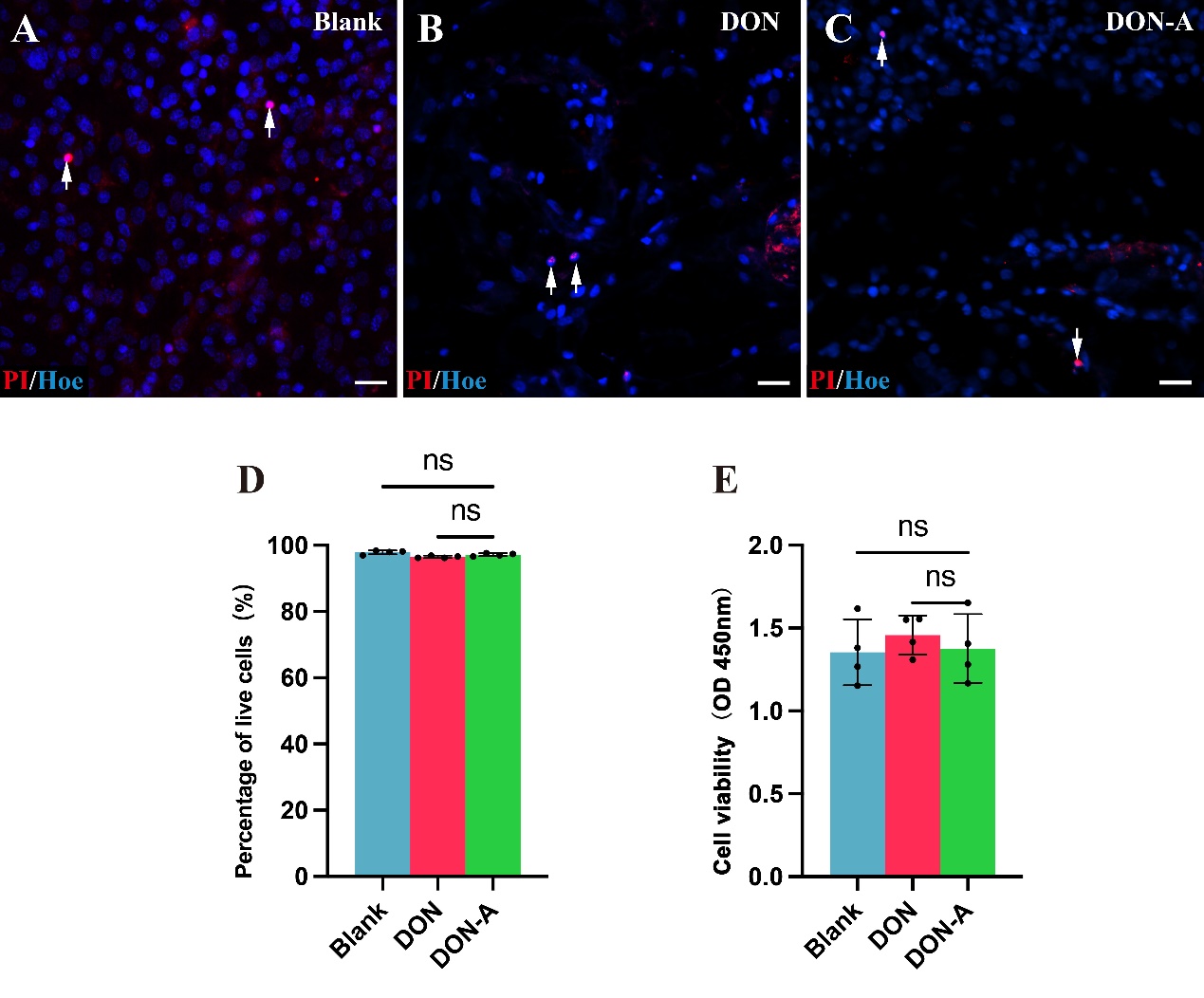


**Figure S4 The survival and viability of NSCs.** (A-C) Survival of NSCs by PI/Hoe, PI positive cells (arrows) indicate the dead cells. (D-E) Bar chart showing the survival and viability of NSCs in the Blank, DON, DON-A group assessed by PI/Hoe (D) and CCK-8 (E) analysis (*n*=4, one-way ANOVA with LSD-t post-hoc test, ns indicates no significant difference). Scale bars = 20 μm in (A-C).


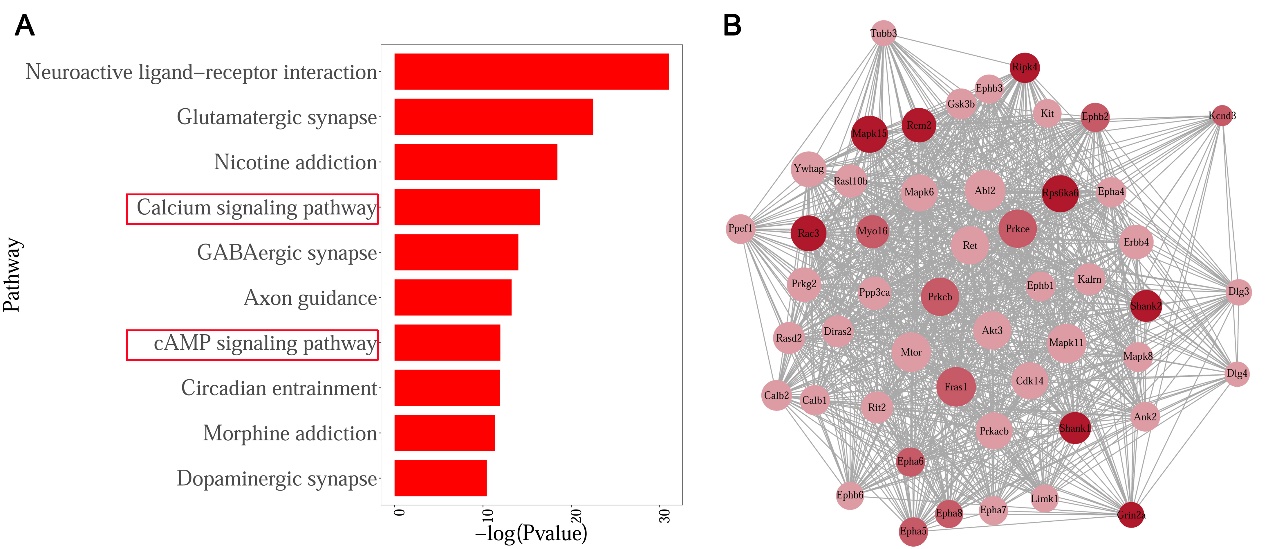


**Figure S5** **KEGG enrichment analysis and protein-protein interaction network analysis.** (A) Bar chart showing the top10 upregulated KEGG enrichment pathway in the DON-A group when compared to the DON group. (B) Protein-protein interaction network analysis of corresponding genes.


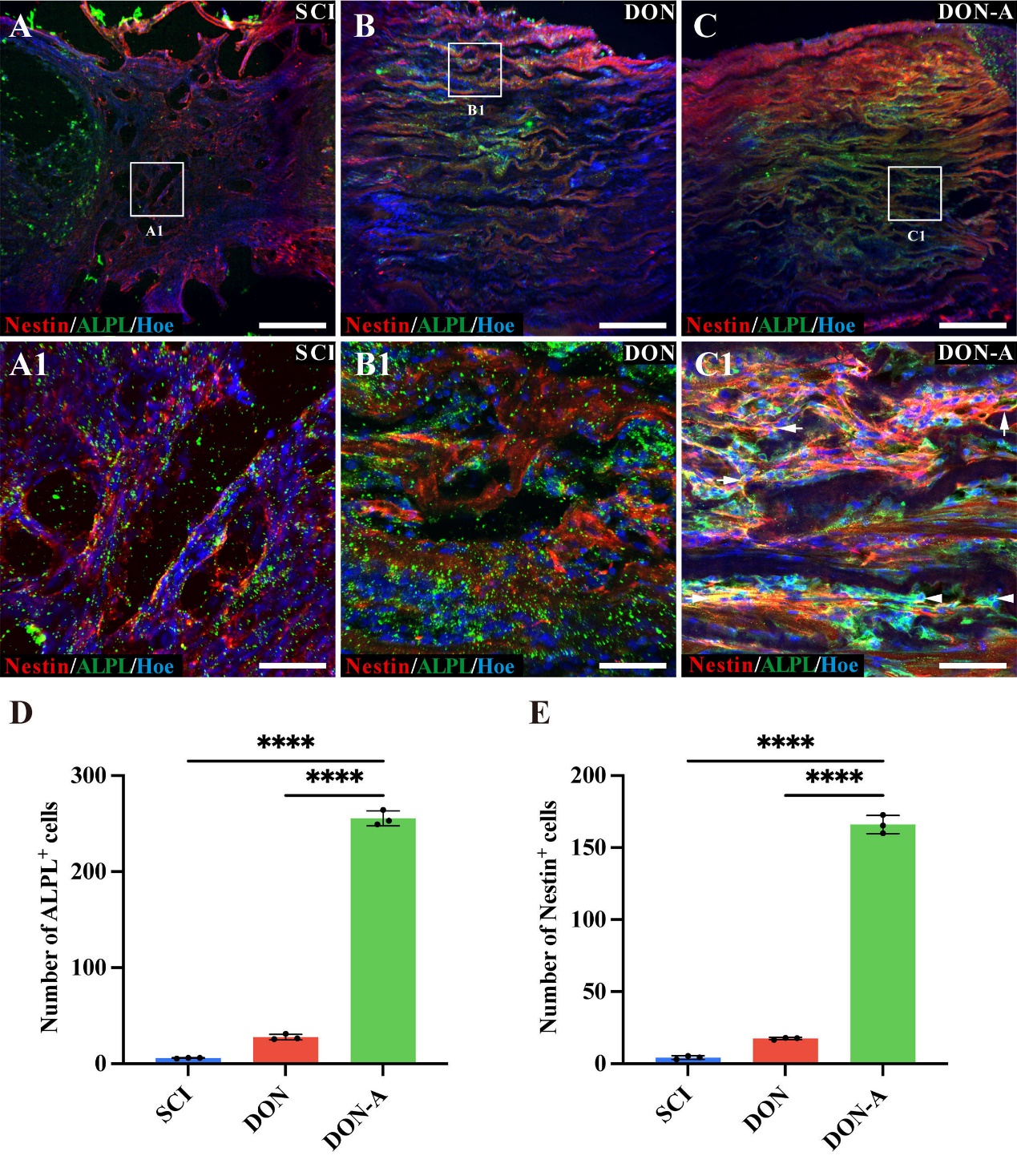
­­­

**Figure S6 Endogenous stem cells recruiting by DON-A *in vivo* two weeks after SCI.** (A, A1) The expression of Nestin and ALPL in the injury site in the SCI group. (B, B1) The expression of Nestin and ALPL in the graft site in the DON group. (C, C1) The Nestin^+^/ALPL^+^ cells (arrows) and ALPL^+^ cells (arrowheads) in the graft site in the DON-A group. (D, E) Bar chart showing the number of ALPL^+^ cells (D) and Nestin^+^ cells (E) in the three groups (n=5, one-way ANOVA with LSD-t post-hoc test, *****P <* 0.0001). Scale bars = 200μm (A-C); 30 μm (A1-C1).


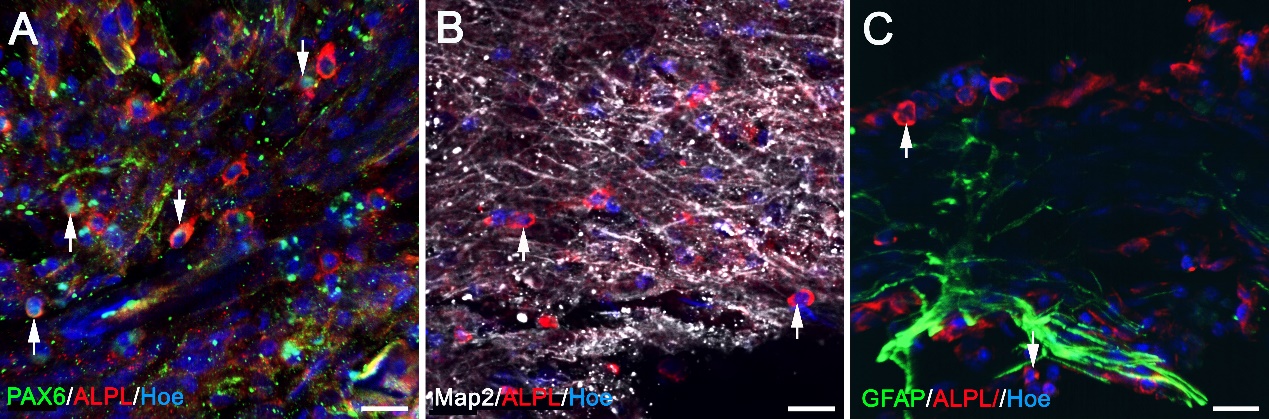


**Figure S7 Detection of ALPL expression on different types of cells.** (A-C) Co-stained ALPL with PAX6 (A, arrows), Map2 (B, arrows) and GFAP (C, arrows) in the graft site in the DON-A groups. Scale bars = 20μm (A-C).


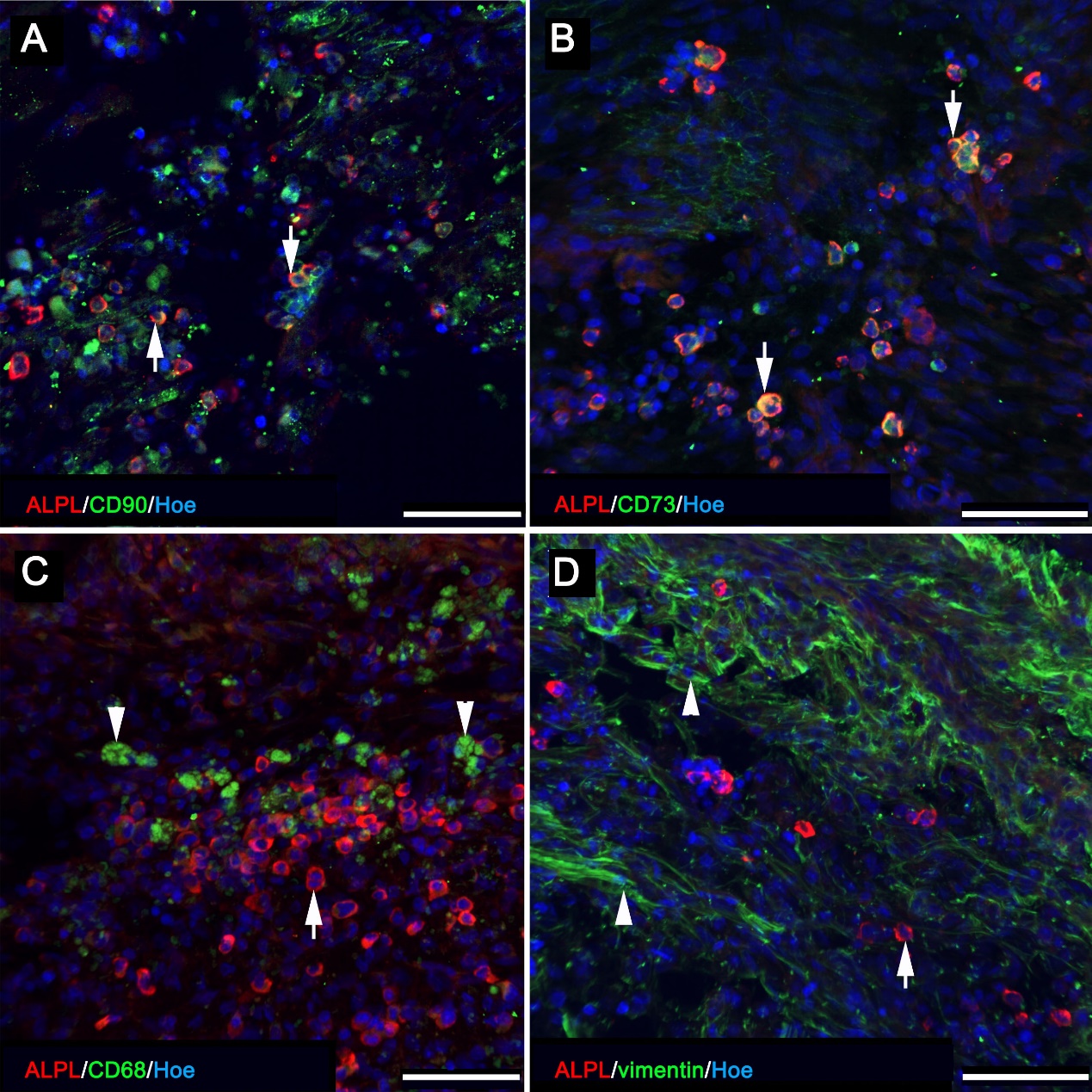


**Figure S8 Detection of** **ALPL expression on different types of cells.** (A-D) Co-stained ALPL with CD90 (A, arrows), CD73 (B, arrows), CD68 (C, arrowheads) and vimentin (D, arrows) in the injury/graft site in the DON-A groups. Scale bars = 30μm (A-D).


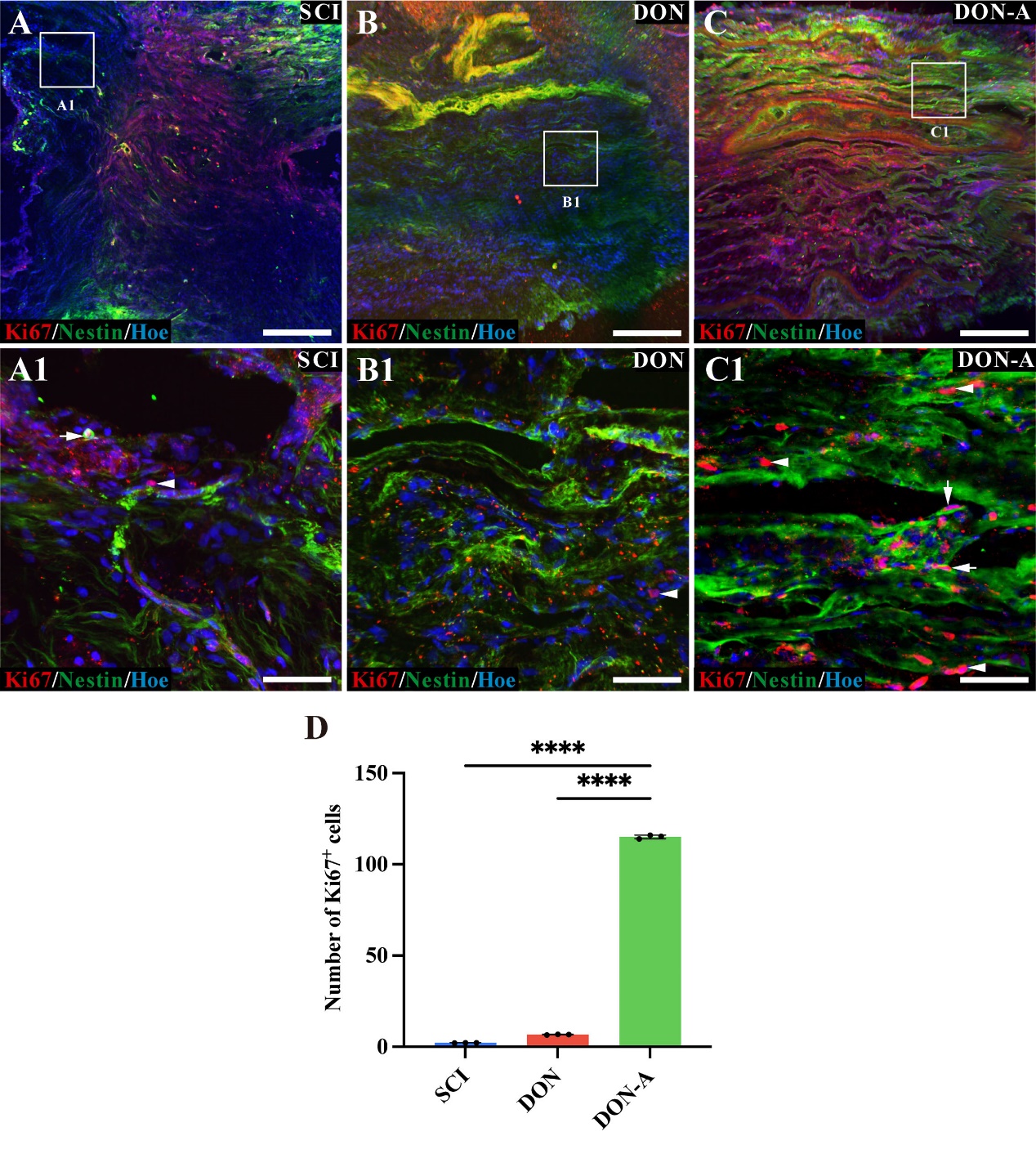


**Figure S9** **NSCs proliferate in situ following recruitment *in vivo* two weeks after SCI.** (A, A1) The expression of Ki67 and Nestin in the injury site in the SCI group. (B, B1) The expression of Ki67 and Nestin in the injury site in the DON group. (C, C1) The Ki67^+^/Nestin^+^ cells (arrows) and Ki67^+^ cells (arrowheads) in the graft site in the DON-A group. (D) Bar chart showing the number of Ki67^+^ cells in the three groups (n=5, one-way ANOVA with LSD-t post-hoc test, *****P <* 0.0001). Scale bars = 200μm (A-C); 30 μm (A1-C1).


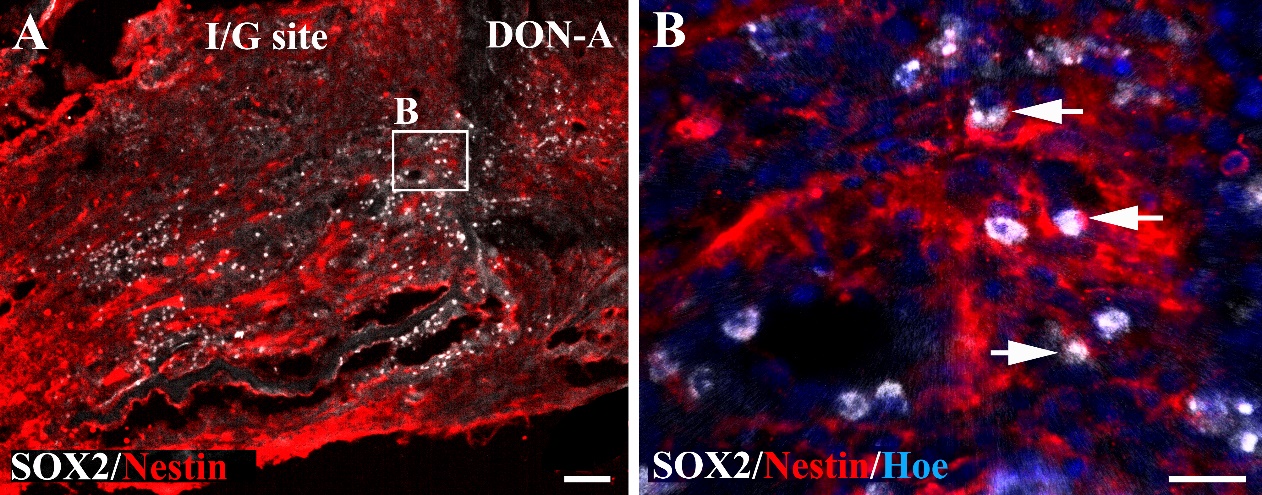


**Figure S10 Endogenous neural stem cells recruiting by DON-A *in vivo* two weeks after SCI.**

(A) Low magnification image of sagittal sections of spinal cord showing the expression of SOX2 and Nestin in the injury/graft site (I/G site) in the DON-A group. (B) High magnification images showing the Nestin^+^/ALPL^+^ cells (arrows) in the graft site in the DON-A group. Scale bars =100μm (A); 20 μm (B).


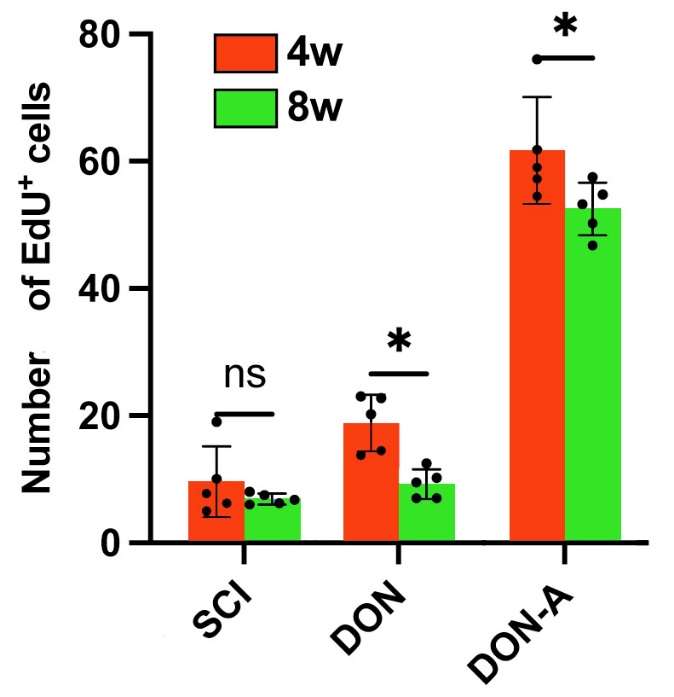


**Figure S11** The number of EdU^+^ cells at four weeks and eight weeks in SCI DON and DON-A groups (*n*=5, one-way ANOVA with LSD-t post-hoc test, *P < 0.05).


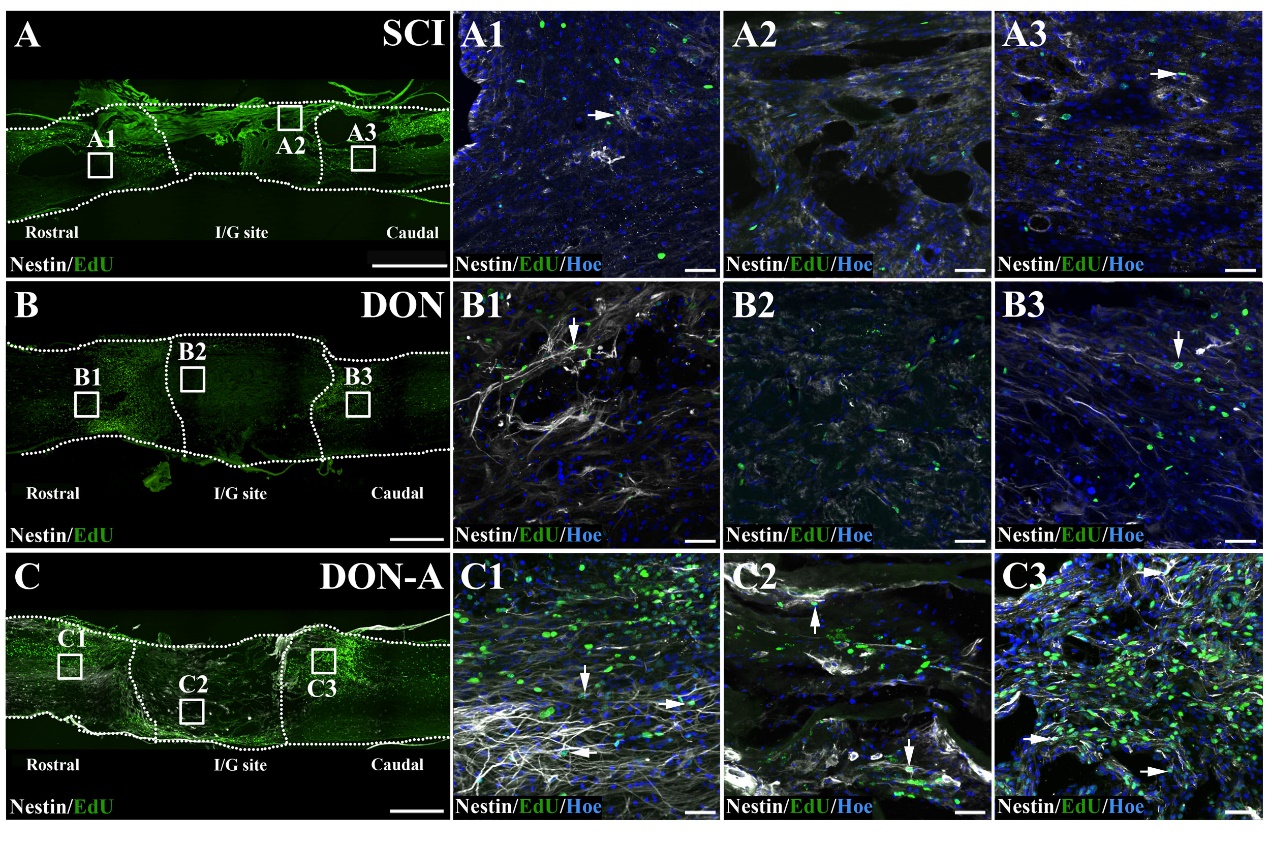


**Figure S12** **Endogenous NSCs colonized in the DON-A scaffolds *in vivo* eight weeks after SCI.** (A-C) Low magnification image of sagittal sections of spinal cord showing the expression of EdU and Nestin in the SCI (A), DON (B) and DON-A (C) groups. (A1-C3) Nestin^+^/EdU^+^ cells in the boundary regions rostral to the injury/graft site (A1, B1, C1, arrows), at the injury/graft site (A2, B2, C2, arrows), and the boundary regions caudal to the injury/graft site (A3, B3, C3, arrows) in the three groups. Scale bars = 1000μm (A-C); 50 μm (A1-C3).


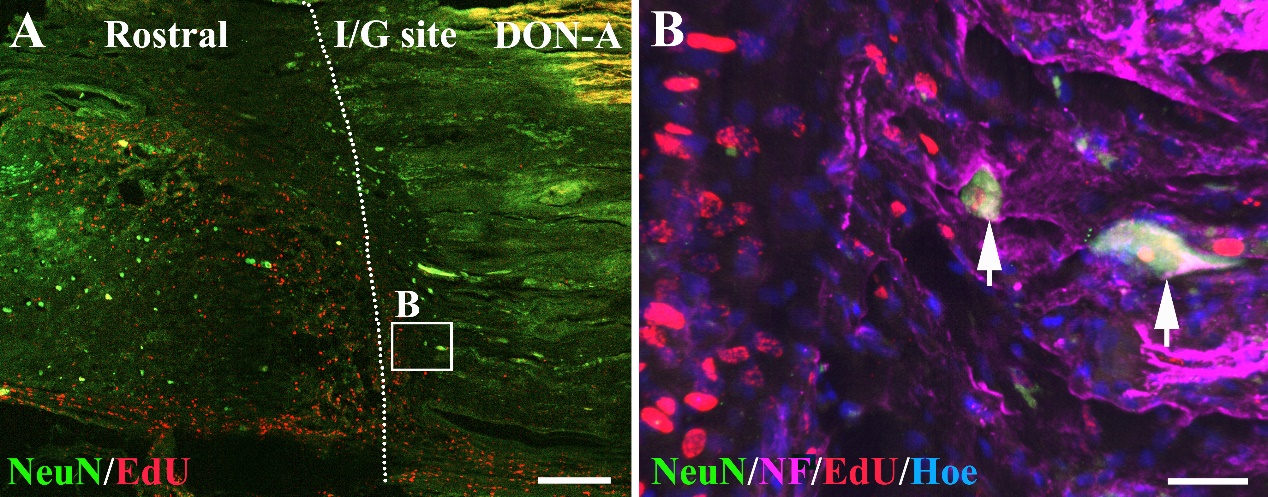


**Figure S13** **Newborn neurons were observed in the DON-A scaffolds *in vivo* at sixteen weeks post-SCI.** (A) Low magnification image of sagittal sections of spinal cord showing the expression of NeuN and EdU in the injury/graft site (I/G site) in the DON-A group, the DON-A scaffold was well-integrated with the spinal cord tissue and retained channels conducive to the linear regeneration of nerve axons. (B) High magnification images showing the NeuN ^+^/NF^+^/EdU^+^ newborn neurons (arrows) in the graft site in the DON-A group. Scale bars =300μm (A); 30 μm (B).

| **Table S1. Primary and secondary antibodies** | | | | |
| --- | --- | --- | --- | --- |
| Antibodies | Species | Type | Dilution | Source (Catalog) |
| Neurofilament 200 (NF) | Chicken | Polyclonal IgG | 1:500 | Abcam, London, UK (ab4680) |
| Synaptophysin (Syp) | Mouse | Monoclonal IgG | 1:200 | Sigma, St. Louis, USA (S5768) |
| Synaptophysin-647 (Syp-647) | Rabbit | Monoclonal IgG | 1:1000 | Abcam, London, UK (ab196166) |
| Microtubule-associated protein 2 (Map2) | Mouse | Monoclonal IgG | 1:1000 | Sigma, St. Louis, USA (M4403) |
| Glial fibrillary acidic protein (GFAP) | Rabbit | Polyclonal IgG | 1:2000 | Abcam, London, UK (ab7260) |
| Nestin | Mouse | Monoclonal IgG | 1:500 | Abcam, London, UK (ab6142) |
| Ki67 | Rabbit | Monoclonal IgG | 1:500 | Abcam, London, UK (ab16667) |
| vimentin | Mouse | Monoclonal IgG | 1:1000 | Abcam, London, UK (ab8978) |
| growth-associated protein (GAP43) | Mouse | Monoclonal IgG | 1:500 | Abcam, London, UK(ab315198) |
| calcitonin gene related peptide (CGRP) | Mouse | Monoclonal IgG | 1:1000 | Abcam, London, UK (ab81887) |
| Myelin basic protein (MBP) | Rabbit | Polyclonal IgG | 1:400 | Merck Millipore, Billerica, USA (AB980) |
| Beta III Tubulin (Tuj) | Mouse | Monoclonal IgG | 1:1000 | Abcam, London, UK (ab78078) |
| Beta III Tubulin(Tuj) | Rabbit | Polyclonal IgG | 1:200 | Sigma, St. Louis, USA (T2200) |
| Alkaline phosphatase | Rabbit | Polyclonal IgG | 1:100 | Elabscience, CN (40142) |
| CD68 | Mouse | Monoclonal IgG | 1:200 | Abmart, CN (MN50019) |
| CD73 | Rabbit | Polyclonal IgG | 1:200 | Abmart, CN (TD6763) |
| CD90 | Mouse | Monoclonal IgG | 1:500 | Abmart, CN (MU142715) |
| CD31 | Rabbit | Polyclonal IgG | 1:200 | Abmart, CN (TA6191) |
| von Willebrand factor (VWF) | Rabbit | Polyclonal IgG | 1:200 | Abcam, London, UK (ab6994) |
| Oligodendrocyte transcription factor 2 (Olig2) | Rabbit | Monoclonal IgG | 1:5000 | Abcam, London, UK (ab109186) |
| SRY-box 2 (SOX2) Conjugate555 | Rabbit | Monoclonal IgG | 1:100 | Cell signaling, USA (5179S) |
| Streptavidin  Conjugate555 |  |  | 1:300 | Invitrogen, USA (S32355) |
| Alexa fluor 488 goat anti-chicken secondary antibody | Goat | Polyclonal IgG | 1:800 | Abcam, London, UK (ab150169) |
| Alexa fluor 488 goat anti-rabbit secondary antibody | Goat | Polyclonal IgG | 1:800 | Abcam, London, UK (ab150077) |
| Alexa fluor 488 goat anti-mouse secondary antibody | Goat | Polyclonal IgG | 1:800 | Abcam, London, UK (ab150113) |
| Alexa fluor 555 goat anti-mouse secondary antibody | Goat | Polyclonal IgG | 1:800 | Abcam, London, UK (ab150114) |
| Alexa fluor 555 goat anti-rabbit secondary antibody | Goat | Polyclonal IgG | 1:800 | Abcam, London, UK (ab150169) |
| Alexa fluor 647 goat anti-mouse secondary antibody | Goat | Polyclonal IgG | 1:800 | Abcam, London, UK (ab150115) |
| Alexa fluor 647 goat anti- rabbit secondary antibody | Goat | Polyclonal IgG | 1:800 | Abcam, London, UK (ab150079) |
| [Goat anti-mouse HRP](http://www.abcam.com/goat-mouse-igg-hl-hrp-ab6789.html) | Goat | Polyclonal IgG | 1:2000 | Abcam, London, UK (ab6789) |
| [Goat anti-rabbit HRP](http://www.abcam.com/goat-mouse-igg-hl-hrp-ab6789.html) | Goat | Polyclonal IgG | 1:2000 | Abcam, London, UK (ab6721) |
